# Local increases in admixture with hunter-gatherers followed the initial expansion of Neolithic farmers across continental Europe

**DOI:** 10.1126/sciadv.adq9976

**Published:** 2025-08-20

**Authors:** Alexandros Tsoupas, Carlos S. Reyna-Blanco, Claudio S. Quilodrán, Jens Blöcher, Maxime Brami, Daniel Wegmann, Joachim Burger, Mathias Currat

**Affiliations:** ^1^ Department of Genetics and Evolution, University of Geneva, Geneva, Switzerland.; ^2^ Swiss Institute of Bioinformatics, Lausanne, Switzerland.; ^3^ Department of Biology, University of Fribourg, Fribourg, Switzerland.; ^4^ Palaeogenetics Group, Institute of Organismic and Molecular Evolution (iomE), Johannes Gutenberg University Mainz, Mainz, Germany.; ^5^ Institute of Genetics and Genomics in Geneva (IGE3), University of Geneva, Geneva, Switzerland.

## Abstract

The replacement of hunter-gatherer lifestyles by agriculture represents a pivotal change in human history. The initial stage of this Neolithic transition in Europe was instigated by the migration of farmers from Anatolia and the Aegean basin. In this study, we modeled the expansion of Neolithic farmers into central Europe along the continental route of dispersal. We used spatially explicit simulations of paleogenomic diversity and high-quality paleogenomic data from 67 prehistoric individuals to assess how population dynamics between Indigenous European hunter-gatherers and incoming farmers varied across space and time. Our results demonstrate that admixture between the two groups increased locally over time at each stage of the Neolithic expansion along the continental route. We estimate that the effective population size of farmers was about five times that of hunter-gatherers. In addition, we infer that sporadic long-distance migrations of early farmers contributed to their rapid dispersal, while competitive interactions with hunter-gatherers were limited.

## Teaser

The first farmers of continental Europe increasingly admixed over time with local hunter-gatherers.

## INTRODUCTION

Paleogenomic studies have revealed that the genetic trajectory of modern Europeans has been strongly shaped by the expansion into Europe of early farmers from the Aegean basin and western Anatolia starting around 8600 years before present (yr B.P.) (*1*–*3*), introducing agriculture in regions that were previously dominated by hunter-gatherer lifestyles (*4*). The question of whether the Neolithic transition was primarily a cultural process, in which hunter-gatherer groups adopted farming from neighboring communities (*5*), or instead a demic process, in which farmers migrated from Southwest Asia into Europe (*6*), has long been discussed, with studies highlighting the regional role of both processes [e.g., (*7*, *8*)]. This long-standing debate decisively shifted toward favoring migration as a key factor following Bramanti *et al*.’s work (*1*), which revealed substantial genetic differences between central European hunter-gatherers and the first agriculturalists. The latter displayed a genetic signature that only emerged in Europe during the Neolithic period. Subsequent genomic studies supported these findings [e.g., (*9*)], successfully tracing the ancestral roots of early Neolithic populations in Europe to the area encompassing Western Anatolia and the Aegean basin (*2*, *3*). It was further established that the early Neolithic inhabitants of present-day Iran differed genetically from Aegean and European farmers (*10*), demonstrating that the Neolithic migration chain did not begin in the eastern wing of the Fertile Crescent (*11*), but rather further west, possibly in the Taurus foothills or on the Anatolian plateau (*2*). Recently, Marchi *et al*. (*12*) presented evidence that the European early Neolithic population originated in Anatolia through a process involving population mixing during the Last Glacial Maximum and genetic drift at the onset of the Holocene. The resulting population adopted agriculture in Anatolia and gradually expanded toward central Europe, eventually reaching Northern and Western Europe (*12*). This dispersal route—proceeding along the Danube, the Rhine, and other major river valleys—is described in the archeological literature as the “continental” or “Danubian route” of Neolithic expansion [e.g., (*7*, *13*)]. It is usually contrasted with the “maritime” or “Mediterranean” route, which followed a coastal path along the Mediterranean shorelines, resulting in the colonization of southern Europe and eventually the settlement of southern France, northern Africa, and the Iberian Peninsula (*14*).

The present study focuses on the continental route of Neolithic expansion. Before the disappearance of hunter-gatherer lifeways in Europe, farmers and foragers coexisted for many generations and in certain instances for thousands of years (*15*). Many questions remain about the biological interactions between the two populations, which spanned more than 3000 years, from the Balkans to Denmark (*16*). Although the general pattern of early farmer migration seems consistent across the western part of Europe, there is regional and temporal variation in the levels of admixture with hunter-gatherers [e.g., (*17*–*19*)], and regional studies provide valuable insights into local population interactions [e.g., (*20*–*22*)]. Some paleogenomic data support the assertion that hunter-gatherers and farmers only admixed sporadically at the outset of the Neolithic (*3*, *23*). Other studies indicate that admixture intensified at later stages of the Neolithic [starting ~7000 yr B.P.; (*18*, *19*, *22*, *24*)], possibly after the so-called “crisis” of the “LBK” (Linearbandkeramik). This period, which spans until the central European Middle Neolithic, is characterized by massacre sites, mass graves, and widespread abandonment of sites (*25*, *26*). Spatially, the levels of hunter-gatherer ancestry in Neolithic populations appear to increase east of the axis that goes from the Black Sea to the Baltic region (*16*). There is no such clear trend from Southern to Northern Europe, although more admixture at higher latitudes was proposed (*19*), possibly linked to a slowdown of the Neolithic expansion speed (*27*). Moreover, Rasteiro and Chikhi (*28*) have described a trend of decreasing female and male genetic lineages of Neolithic farmers in the genome of modern Europeans, with distance from the Middle East, an observation compatible with the demic diffusion model (*6*) including only very limited local hunter-gatherer introgression into migrating farmers at each step of their progression (*29*, *30*).

Here, we wanted to better understand the population dynamics underlying the interactions between hunter-gatherers and farmers along the continental route of the European Neolithic. We modeled the Neolithic expansion by jointly simulating demography, migration, and biological interactions (in the form of admixture and competition) between two populations, within a spatially explicit framework. We then used an approximate Bayesian computation [ABC; (*31*, *32*)] framework to infer key demographic parameters by comparing the resulting simulated paleogenomic data to recently published high-quality genomic data from 67 individuals. Compared to previous demogenomic modeling of the Neolithic using ancient DNA, our simulation framework is able to manage more explicit spatial features than Marchi *et al.* (*12*) and to handle the spatio-temporal variation of admixture between hunter-gatherers and farmers with more flexibility than Rasteiro and Chikhi (*28*) and Silva *et al.* (*33*). The paleogenomic dataset we have used is particularly well suited for demogenomic inferences as it is composed of genome-wide sequences selected from presumably unlinked and neutral regions (*34*). Moreover, their high sequencing quality allowed us to directly compute molecular indices of intergenomic diversity (i.e., nucleotide diversity) rather than summarizing the molecular information through estimated admixture proportions from putative source populations [e.g., (*35*)]. We specifically examined what dynamics characterized the demographic processes underlying the interactions between hunter-gatherers and farmers and whether variations in admixture between these populations are detectable over time and space.

## RESULTS

### Model choice among six demographic scenarios

**Fig. 1.**
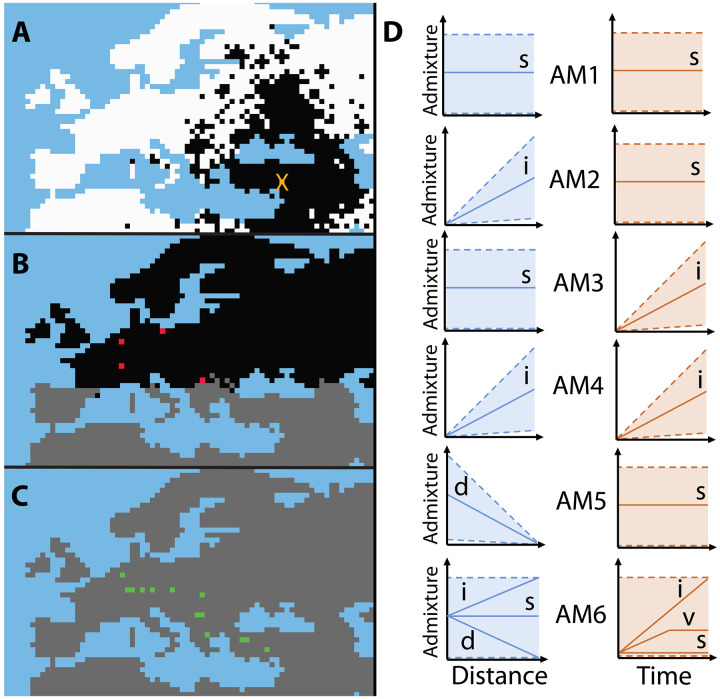
Graphical representation of spatially explicit simulations and investigated scenarios. On the maps: Blue, water cells; white, cells with only Hunter-Gatherers (HG); gray, cells with only farmers (FA); black, cells with both HG and FA. (**A**) Example of simulation with the spread (including long-distance dispersals) of FA from the orange cross toward northwestern Europe through the continental route. (**B**) Two zones with different competition coefficients, delimited by latitude 43.20° (α_S_: competition coefficient in the southern zone; α_N_: competition coefficient in the northern zone). In the gray zone (below latitude 43.2°), α_S_ = 1, while in the black zone (above latitude 43.2°), α_N_ is variable (taken from a prior distribution), allowing a longer cohabitation between HGs and FAs; red cells show demes where HG samples are simulated. (**C**) At the end of the simulation, HGs disappear from the whole simulated area; green cells show demes where FA samples are simulated. (**D**) Schematic illustration of the differences of the investigated scenarios. Solid lines represent the possible trends and dashed lines represent the possible variation of the admixture rate γ. γ between HGs and FAs can be stable (s), increase (i), or decrease (d) with distance from the starting point of the FA expansion in Anatolia (blue) and be stable in time (s) or increase during the whole cohabitation period (i) or increase during a variable time within the cohabitation period (v) (orange).

We started by comparing six variations of a model (thereafter called scenarios and named AM1 to AM6), in which admixture with Indigenous hunter-gatherers (HG) varied through space and/or time when Neolithic farmers (FA) migrated from Anatolia toward Europe. The admixture rate in the investigated scenarios was constant or increasing in time, while it was constant, increasing, or decreasing with distance from Anatolia (see Fig. 1D for a schematic illustration of the six scenarios). For scenarios AM1 to AM5, the admixture rate increases during the entire period of HG-FA cohabitation, whereas in AM6, the period of increase is variable and corresponds to a proportion of the cohabitation period. It is worth noting that in all scenarios, only a relatively small proportion of simulations (~4%) were able to replicate the entire dataset of samples, i.e., that all sampling locations are populated by the corresponding population (HG or FA) at the time estimated for the empirical data. The proportion of rejected simulation is quite similar among scenarios, going from 95.5% (AM3) to 95.8% (AM2 and AM6). The number of simulations available for the ABC estimation is thus limited despite intensive computation, and we therefore used the ABC-random forest approach for model choice, as text S1 demonstrates that using only 13,000 simulations per scenario is enough to distinguish the most similar scenarios when summarizing the genomic information into pseudo-haploid genotypes. All six investigated scenarios were able to reproduce the observed molecular diversity (table S1), as shown by marginal density *P* values larger than 0.05 and were therefore retained for further analysis.

The most probable scenario among the six was AM6 (table S2), with a posterior probability of 0.77. This suggests that the observed levels of genetic differentiation between HGs and FAs are more accurately described by a scenario in which the rate of admixture between the two groups increases locally over time, for a variable time period.

Given that individual assignment to the HG or FA groups can affect the results, we chose to rerun simulations removing genomes from the exceptional site of Lepenski Vir in Serbia, in which the group attribution is especially challenging (*20*, *21*). However, this does not change model choice, with model AM6 still being the best with a relative probability of 0.70 (text S2).

**Fig. 2.**
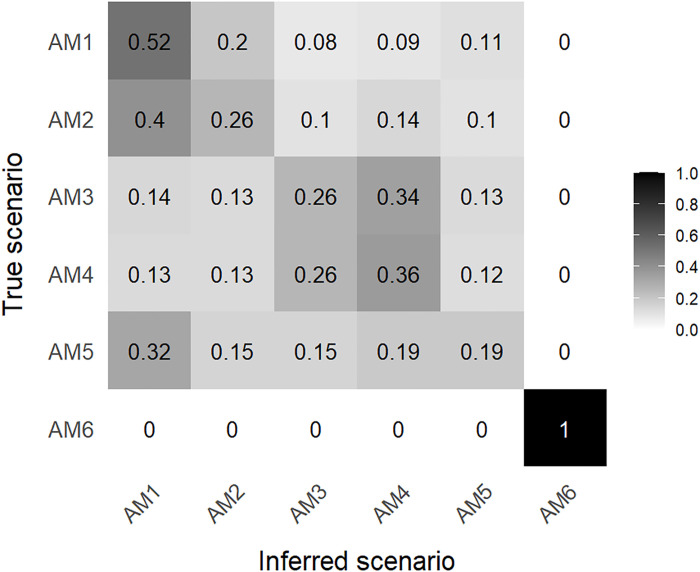
Confusion matrix for the six investigated scenarios. Calculation performed with abcrf R package [random forest approach (*80*)] using 33,500 simulations per scenario. The numbers correspond to the proportion of simulations that was assigned to each case by ABC. Each row represents simulations from a given scenario (the ‘true’ scenario), while the columns indicate the proportion of those simulations that ABC attributed to each of the studied scenarios (the inferred scenarios). For the analysis, the untransformed pairwise values of inter- and intrasample pseudo-haploid nucleotide diversity were used.

From the confusion matrix (Fig. 2), we observe that only scenario AM6 can be clearly differentiated from the other ones. Among the other scenarios, those with spatially increasing admixture (i.e., higher admixture rate in the northwest of the continental route than in the southeast) cannot be differentiated well from the ones where admixture is constant in space (AM2 and AM4 from AM1 and AM3, respectively). However, scenarios in which admixture increases temporally in each deme since their first colonization are easier to differentiate from those in which admixture is constant over time (AM3 and AM4 from AM1 and AM2). Scenario AM5, in which admixture is higher in the southeast than in the northwest of the continental route, produces a pattern of diversity not distinguishable from the other five scenarios, as its simulations are randomly classified among them. Performing the model choice with only scenarios AM1 to AM5 to examine their relationships in more detail (text S3), we see that the most probable scenario is AM3 [posterior probability of 0.43 (see table S6), with a 0.26 probability to be correctly identified (see fig. S6)], where the admixture rate increases locally with time, a characteristic that AM3 shares with AM6. Seeing that scenario AM5 is misclassified more often than correctly identified and that scenarios AM3 and AM4 are hardly distinguishable, we performed another model choice using a subset of only the three first scenarios (AM1, AM2, and AM3) to avoid noise (text S4). In this case, the most probable scenario is AM3 again, with the posterior probability increasing to 0.52 (table S7) and a 0.66 probability of being correctly identified (fig. S7).

### Parameter estimation using a single demographic scenario

The most probable scenario is thus AM6, representing the admixture rate increasing with time in each deme, but with a nuance compared to the other scenarios: In AM6, the duration of the increase of the admixture rate varies within the cohabitation period between HGs and FAs (in contrast to AM3 and AM4’s constant increase over the whole cohabitation period). This allows us to estimate the duration of the admixture rate increase (see below).

**Fig. 3.**
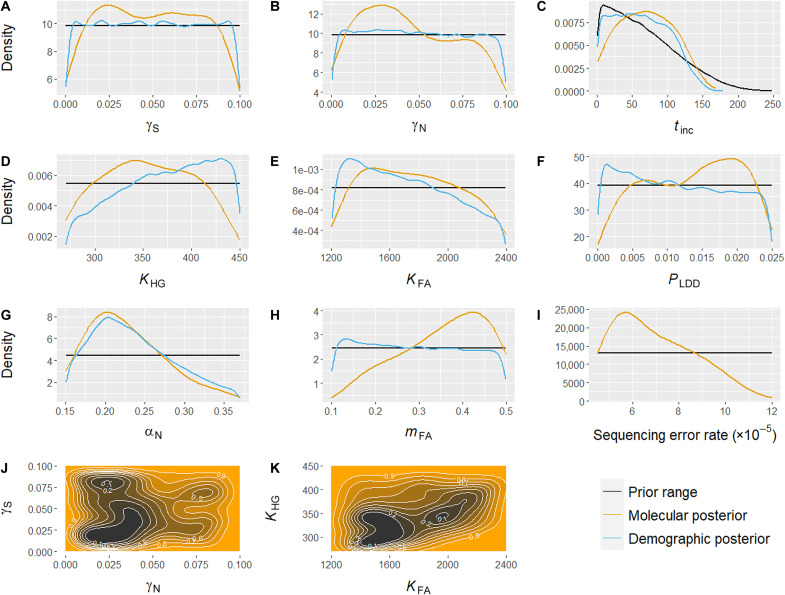
Distribution of the estimated parameter values. In (A) to (I), the black line corresponds to the prior distribution, the light blue one to the distribution of parameter values of the simulations that were able to reproduce the observed dataset (demographic posterior), and the orange line corresponds to the posterior resulting from the ABC estimation (molecular posterior). (**A**) Admixture rate along the southeast continental route (γ_S_); (**B**) admixture rate along the northwest continental route (γ_N_). (**C**) Number of generations during which the admixture rate increases (*t*_inc_). (**D**) Effective population size of HGs (*K*_HG_); (**E**) Effective population size of FAs (*K*_FA_). (**F**) Proportion of FA migrations that are long-distance dispersals (*P*_LDD_). (**G**) Competition coefficient along the northwest continental route (α_N_). (**H**) Migration rate of FAs (*m*_FA_). (**I**) Sequencing error rate (ε). (**J**) Two-dimensional posterior distribution of admixture rate (γ) along the southeast continental route (γ_S_) against the admixture rate along the northwest continental route (γ_N_). (**K**) Two-dimensional posterior distribution of effective population size (*K*) of HGs (*K*_HG_) against the effective population size of FAs (*K*_FA_). For the two-dimensional posterior distributions (J and K), orange represents the value combinations with lower probability and black the combinations with higher probability.

About 96% of the simulations of AM6 were rejected due to what we call here a “demographic filter,” meaning that they were not able to reproduce the whole dataset (e.g., populations existing in all demes where and when empirical samples were collected, see Materials and Methods). The marginal density *P* value for the remaining simulations under AM6 (*P* = 0.29 for δ = 0.01, *P* = 0.32 for δ = 0.005, and *P* = 0.18 for δ = 0.05), indicates its capability to reproduce the observed values of molecular diversity. The characteristics of the posterior distributions of the estimated parameters are presented in Table 1 (with δ = 0.01, while results for δ = 0.05 and δ = 0.005 are presented in table S8), while a graphical representation of the posterior and prior distributions of each parameter is given in Fig. 3. For all parameters except the sequencing error rate, we plotted in addition the distribution of parameter values of the simulations retained by the demographic filter (see light blue lines in Fig. 3). We call it demographic posterior to differentiate it from the posterior that results from the ABC estimation made on molecular statistics, which we call here “molecular posterior” (see orange lines in Fig. 3). The demographic posterior reflects the parameter values that most frequently produce simulations able to generate the whole dataset. For instance, simulations with higher hunter-gatherer effective population size, within the prior range, tend to more often reproduce the observed data (Fig. 3D). For the two γ variables (γ_S_ and γ_N_) and the two *K* variables (*K*_HG_ and *K*_FA_), we estimated two-dimensional posteriors and plotted the estimated high density intervals (HDIs) (Fig. 3) to investigate if there are combinations of these variables that are more probable than others.

**Table 1.** Characteristics of the posterior distributions of the estimated parameters of scenario AM6. We performed 100,000 simulations with 0.01 tolerance level, retaining 1000 simulations. The estimation was performed with ABCtoolbox2 (*81*).

Parameter	Posterior mode	Posterior mode relative bias	Posterior mean	Posterior mean relative bias	Posterior lower 95% HDI	Posterior upper 95% HDI
Admixture rate in southeast continental route (γ_S_)	0.024	3.96	0.049	3.67	0.003	0.096
Admixture rate in northwest continental route (γ_N_)	0.029	4.38	0.046	3.77	0.002	0.093
Number of generations of γ increase (*t*_inc_)	70	1.49	72	2.29	2	136
HG effective population size (*K*_HG_)	343	0.14	357	0.12	273	435
FA effective population size (*K*_FA_)	1479	0.18	1769	0.17	1230	2322
Migration rate of FAs (*m*_FA_)	0.4232	0.40	0.344	0.37	0.162	0.5
Proportion of long-distance dispersals (*P*_LDD_)	0.0192	2.26	0.013	3.50	0.002	0.024
Competition coefficient in northwest continental route (α_N_)	0.2033	0.17	0.231	0.19	0.15	0.327
Sequencing error rate (ε)	0.000057	0.22	0.00007	0.22	0.00005	0.0001

The parameter estimation with scenario AM6 does not detect any spatial variability in the admixture rate. γ in both parts of the continental route is characterized by wide, overlapping posterior 95% HDI (γ_S_ = [0.3 to 9.6%] and γ_N_ = [0.2 to 9.3%]), with posterior modes at 2.4 and 2.9%, respectively (Table 1). The mean and mode are characterized by large relative biases (Table 1), which implies that these point estimates are not accurate. The two-dimensional posterior did not indicate any particular combination of γ_S_ and γ_N_ being more probable than others (Fig. 3J). From the two-dimensional posterior, we estimated that γ_S_ being larger than γ_N_ and the reverse are equally probable (probabilities of 0.48 and 0.52, respectively). This aligns with the model comparison based on the subset of five scenarios (AM1 to AM5), where scenarios differing only in the spatial variability of the admixture rate were not well distinguished (fig. S6). Specifically, scenarios sharing the same temporal trend but differing spatially, such as AM1 with AM2 and AM3 with AM4, were frequently misclassified as one another. This suggests that the spatial pattern of admixture rate cannot be confidently supported or ruled out. Moreover, we estimated that the admixture rate between HGs and FAs was increasing for about 70 generations on average (~1750 years), with the values varying between 2 and 136 generations (~50 to 3400 years), the point estimate being relatively imprecise (Table 1). The observation that the admixture rate increased locally over time is further supported by the model selection restricted to scenarios AM1 to AM5, where scenario AM3, characterized by a temporally increasing admixture rate, was identified as the most probable, with a posterior probability of 0.43 (table S6).

The effective population sizes (*K*, in number of chromosome copies) have broad posterior 95% HDI compared to the prior ones (Table 1). However, the mean and mode relative biases are low, showing relatively accurate means of 357 gene copies for HGs and 1769 for FAs within each deme. Also, the posterior distribution of *K*_FA_ (Fig. 3E) is not uniform but skewed toward lower values. Based on the two-dimensional posterior (Fig. 3K), we estimated that the ratio of the effective population sizes (*K*_FA_*/K*_HG_) ranges between three and seven times larger in favor of FAs, with a probability of 0.96 and a mean of five times.

Our findings indicate that the presence of a low level (~2%) of long-distance dispersals (LDDs) provides a better explanation of the data. This suggests that sporadic LDD events play a role in managing the rapid Neolithic expansion. When using shorter LDD events, on average, of 300 km (instead of 800 km), the marginal density *P* value of the simulated statistics is lower than 0.05, which means that the scenario is no longer able to reproduce the observed summary statistics. In addition, under a scenario without LDDs, only 0.03% of the simulations passed the demographic filter. Together, these observations suggest that sporadic LDD events are needed to cope with the fast Neolithic expansion and for developing the observed pattern of genetic diversity measured in our study. Nevertheless, the impact of their frequency on the measured genetic diversity in our study appears to be limited as the point estimate lacks precision (wide posterior 95% HDI relative to the prior distribution and large relative bias).

We estimated the competition coefficient α_N_ to range approximately from 15 to 33%, a narrower range compared to the original 0 to 100% prior range (text S5). Both the mode and mean values are estimated to be around 20 to 23%, demonstrating a low relative bias (Table 1) and thus indicating accurate estimation of this parameter. The posterior distribution of the sequencing error rate points to a mode value of 5.7 × 10^−5^ with low relative bias, corresponding to a Next Generation Sequencing (NGS) quality score of 42. Even after excluding genomes from the site of Lepenski Vir from the analysis, for which the group attribution is challenging, the parameter estimation remains largely unchanged, confirming the robustness of our results (text S2).

## DISCUSSION

### Local temporal increase in the admixture between HGs and FAs

The demographic modeling approach presented here underscores a nuanced demic diffusion model for the Neolithic spread from Northwest Anatolia extending through the Balkans into central Europe and yields demographic parameter estimates. These findings corroborate a temporal dynamic in admixture between hunter-gatherers (HG) and farmers (FA), indicating a gradual rise in genetic exchange within each location (i.e., deme) over successive generations of cohabitation between these groups. Our results thus suggest that the resurgence of HG ancestry in Neolithic populations after ~7000 yr B.P. (*18*, *19*, *22*) is best explained by local increases in admixture between HGs and FAs over time, rather than a constant accumulation of HG ancestry. It should be noted that our model does not test admixture pulses (*17*), defined as two periods of admixture separated by a period of genetic isolation (*36*), which could be a future development of our approach. We used a linear increase in admixture as a practical proxy to capture the overall temporal dynamics of this parameter. However, we could not determine whether this variation occurred in discrete steps, which would require higher temporal data resolution and the inclusion of additional parameters, such as the number, timing, duration, and intensity of pulses.

It is important to emphasize that the estimated admixture rate is not equivalent to the proportion of HG ancestry in Neolithic populations. Equating the observed proportion of HG ancestry in farming populations with the frequency of admixture can result in misconceptions about historical interactions, as demonstrated by simulation studies (*30*). Instead, the inferred admixture rate offers a more precise measure of the actual interactions between these groups. For instance, theoretical simulations have shown that a low admixture rate (e.g., 5%) at each stage of the Neolithic expansion can lead to a large HG ancestry (e.g., 50%) due to the cumulative effect of population migration, demography, and biological interactions (*30*).

Our estimates show that admixture increased over time, but its magnitude remained low. This ranges from no gene flow upon arrival of farming communities in a local population (deme) to ~5% (with a maximum of 10%) of interactions between HGs and FAs, leading to admixture during later phases of the Neolithic. The “intensity” of admixture—the 95% HDI of the rate we estimated (0.2 to 9.3%)—is of the same order as the values (2 to 7%) of HG incorporation in the first farming communities estimated under a different (not spatially explicit) model by Marchi *et al*. (*12*). Moreover, it falls within the range (<10%) estimated with spatially explicit simulations by Silva *et al.* (*33*) based on ancient mitochondrial DNA and is compatible with the estimation made from autosomal SNPs by Silva *et al.* (*37*) (mean of ~8.8%, 90% HDI = 5 to 14%). We refrained from further comparisons to previous studies, given (i) the differences between the datasets analyzed [>320 samples for a single sex-specific locus in (*33*) and two whole genomes in (*37*)], (ii) the difference between the implemented admixture model [constant admixture in (*37*) and (*12*) but variable in (*33*) and here], and (iii) the limited precision of our admixture rate estimation (wide posterior distribution). This relatively low precision may be due in part to the stochastic effect of LDDs, which has been shown to diminish introgression of local (e.g., HG) alleles during (e.g., FA) range expansions (*38*).

Our results suggest that early farming communities quickly settled into favorable environments suited for agriculture, potentially occupying distinct spatial territories from HG groups (*4*, *39*). This spatial and possibly cultural separation likely restricted interactions between these groups. Subsequently, as agricultural practices stabilized, acculturation or genetic exchanges with external HGs became more intensive. While we have simulated a model with a gradual increase in admixture for simplicity, this approach represents a general trend rather than the actual mode, which may have been more irregular or episodic. The “crisis” in LBK farming communities around 7000 yr B.P., which resulted in a substantial population reduction (*40*, *41*), may have contributed to a steady increase in the genetic contribution of hunter-gatherers to local farming communities. The delayed admixture scenario, supported by our results, draws attention to what prehistorian Stuart Piggott once described as “secondary Neolithic” cultures (*42*). These cultures, initially identified in Western Europe based on continuities in chipped stone traditions, are traditionally thought to represent the assimilation of Neolithic features by hunter-gatherer-fishers after the first impact of the arrival of immigrant farmers in the region (*42*).

Our model does not allow the identification of specific cultures or factors influencing this increased assimilation, whether they are ecological, economic, or climate related (*40*, *43*). Moreover, it remains unclear whether increased interactions between foragers and farmers led to widespread adoption of Neolithic practices, as assumed by traditional frontier expansion models in archeology, such as Marek Zvelebil’s three-stage “availability” model (*44*). We estimated the increase of admixture to have lasted a long time period (~1750 years), consistent with the prolonged coexistence estimated in some parts of central and northern Europe (*15*, *45*). Our estimation tends, however, to be longer than the admixture time of 500 to 840 years estimated for Hungary and 280 to 700 years for Germany (*19*).

### No indication of spatial variation in the admixture between HGs and FAs

Unlike the temporal aspect, our findings do not reveal spatial variability in the admixture rate between HGs and FAs along the continental Neolithic route. The admixture rates estimated in the northwest and in the southeast of the continental route could not be differentiated, with largely overlapping 95% HDI (Table 1), while the two-dimensional posterior indicated that the admixture rate was equally likely to be higher in either region, with no clear preference for one over the other. Moreover, neither a scenario suggesting a higher admixture rate in the northwestern part of the continental route (AM2), nor one indicating a lower rate (AM5), corresponds better to the observed data than a scenario with an equal rate (AM1). Even if it aligns with previous demogenetic modeling studies that relied on a spatially homogeneous admixture rate at the continental scale (*28*, *30*, *46*, *47*), this is in contrast to the hypothesis posited by Betti *et al*. (*27*), which proposes a higher admixture rate in the northwest. However, our cross-validation analysis revealed that our study is better suited to identify temporal variation in admixture than spatial variation, for which we may lack power. Distinguishing between equivalent scenarios, with or without spatial increase in admixture (AM1 versus AM2 versus AM5 and AM3 versus AM4 in Fig. 2), remains for instance challenging. Our dataset may be too limited in spatial coverage to reliably identify geographical variations. Our approach has been tailored to identify overarching spatial trends along the continental route. However, a Neolithic expansion model based on radiocarbon dates has highlighted regional variations in the demographic or cultural diffusion of the Neolithic (*7*). So, we acknowledge the possibility that our analysis may not capture nuanced regional variations. Disentangling spatial and temporal variation in admixture may be hindered by the correlation between genome age and distance from Anatolia (Pearson’s *r* = −0.42, *P* = 0.0004): Older FA genomes are from regions closer to Anatolia, while younger ones are from central Europe. This pattern, inherent to the dataset due to the southeast-to-northwest progression of the Neolithic expansion, may confound spatial and temporal signals. A larger, spatially, and temporally balanced dataset could theoretically help to better decouple age and location, enabling clearer insights into the spatial dimension of admixture variation.

### Demic diffusion with sporadic long-distance migrations

Our results support the view that most of the migration movements occurred in a stepping-stone manner, with a very small proportion involving long-distance migration (LDD), contributing to the rapid advance of the Neolithic expansion front along the continental route in the Balkans and central Europe [e.g., (*27*, *48*)]. LDD is rare but essential, as only 0.03% of simulations without LDD successfully reproduce the full dataset at the locations and dates of the empirical samples. This is in accordance with two modeling studies based on radiocarbon dates in the Central Balkans, supporting a rapid demic diffusion model of expansion with sporadic leapfrog migration events (*49*), possibly including some LDD occurring behind the expansion front with groups of migrants traveling between 88 and 150 km before settling down (*50*). In comparison, we set the average LDD distance in our study to about 800 km, in order to contrast their effect with short-scale migrations that occurred 100 km away (the size of a deme) and because a shorter LDD distance of about 300 km is not able to reproduce the molecular diversity in our dataset, having marginal density *P* values <0.05.

The detection of sporadic LDD in our model contrasts with results from Marchi *et al*. (*12*), who did not find a signal for LDD along the continental route. However, both results are not necessarily incompatible, given the very low proportion of LDD migrations we estimated in our study, which has a much higher spatial resolution. The simulations performed by Marchi *et al*. (*12*) used a much lower number of demes (<10) compared to ours (>100 between the most distant samples), and it is harder to detect rare long-distance events of migration at lower spatial resolution. Together, our results support the view that the Neolithic expansion along the continental route consisted of sporadic LDDs similar to the “leapfrog colonization” model suggested by archeologists (*8*, *51*), with short-scale migrations making up the bulk of the demic diffusion pattern.

### Limited population competition and population effective size ratio

Studies taking into account interpopulation competition between HGs and FAs remain rare. While prior modeling studies integrated competition between HGs and converted FAs using the “wave of advance” model (*52*, *53*) and competition between HGs and “initial” FAs (*54*, *55*), they primarily focused on how competition affects the speed of the advancing FA wave. None of these studies attempted to quantify competition based on molecular data, as we have done in our research. We estimated that a low competition rate between HGs and FAs is necessary to reproduce the long cohabitation period between them in central Europe (*15*, *45*), together with a specific ratio of population sizes for the two groups of ~5 fold. We estimated a coefficient of competition of ~20% under a Lotka-Volterra model, which could be interpreted as both populations occupying partially overlapping niches, i.e., by exploiting about one fifth of resources in common. Some potential explanations for the relatively low competition could be that the early migrating FAs occupied areas not inhabited by HGs (*39*) or that the arrival of FAs in central and northern Europe affected the yield of their crops and therefore their competitiveness (*27*).

In combination with the limited competition rate, we estimated the effective population size of FAs to be between three and seven times higher than that of the HGs. This ratio is within the range used in Silva *et al*. (*33*) and smaller than the value (10× to 30×) assumed in previous simulation studies of the Neolithic transition (*6*, *30*, *46*, *47*).

### Model adaptations and limitations

The spatially explicit simulator, SPLATCHE3, had to be adapted to the specificities of the paleogenomic dataset analyzed. In particular, we incorporated a sequencing error rate in our simulations, as it may bias the inferences when not taken into account (*56*). The posterior distribution points to a sequencing error rate of 5.7 × 10^−5^, which is lower than the rate typically assumed for NGS [around 1 × 10^−3^ (*57*) to 2.4 × 10^−3^ (*58*)] and within the range estimated for NGS data after computational correction [1 × 10^−5^ to 10 × 10^−5^; (*59*)]. This low estimated error rate was expected because we calculated pairwise distances between pseudo-haploid genome calls and only at sites covered at least twice after trimming away bases potentially affected by postmortem damage.

Although our modeling framework is a step toward considering nonuniform demic models of Neolithic expansion by varying admixture rates between HGs and FAs at different latitudinal stages of the continental route, there are still some missing features that would be worth investigating in the future. Firstly, while our model uses logistic population growths for both HGs and FAs, we assumed that their carrying capacities were uniform and constant over both time and space. We thus did not consider the inferred fluctuations in FA population sizes during the Neolithic (*41*, *60*, *61*), nor the differential occupation of diverse environments by both Mesolithic HGs (*62*, *63*) and Neolithic FAs (*51*, *64*). Secondly, by using uniform and constant migration rates, we did not consider arrhythmic leaps in the Neolithic spread (*65*) and potentially faster spread along the coastlines and rivers (*66*) or potentially slower spread over geomorphological barriers such as mountain ranges. It is conceivable, however, that these arrhythmias would have a larger impact on spatial molecular patterns than on the temporal pattern we identified in our study. We assume that arrhythmias would have a greater influence on admixture during the early phases of the expansion, driven by regional disparities, than on the overall long-term trend of increasing admixture during the cohabitation period, but it remains to be tested. Furthermore, incorporating the estimated age range for each observed genome instead of relying solely on mean values could enhance the accuracy of the demographic filter, particularly concerning parameters associated with population movement. Despite leveraging a high-quality paleogenomic dataset for demographic inferences, our study faced hurdles in differentiating scenarios and attaining accurate parameter estimates. These challenges stemmed from the potential of similar outcomes arising from different combinations of parameter values (equifinality issue), alongside information loss during data conversion and dimensionality reduction. These complexities highlight the delicate balance and trade-offs inherent in extracting information about past population dynamics from ancient DNA.

Our simulation framework establishes a foundational platform for the future development of more detailed scenarios to study how population demographic variations, migrations, and interactions affected spatio-temporal patterns of genomic diversity. As paleogenomic data continue to accumulate with improved sequencing quality, this iterative process can markedly enhance our understanding of ancient population dynamics. This will enable more refined demographic estimates regarding not only competition and admixture along the Neolithic continental route but also other major events that have affected the history of our ancestors at different periods.

## MATERIALS AND METHODS

### Dataset

**Fig. 4.**
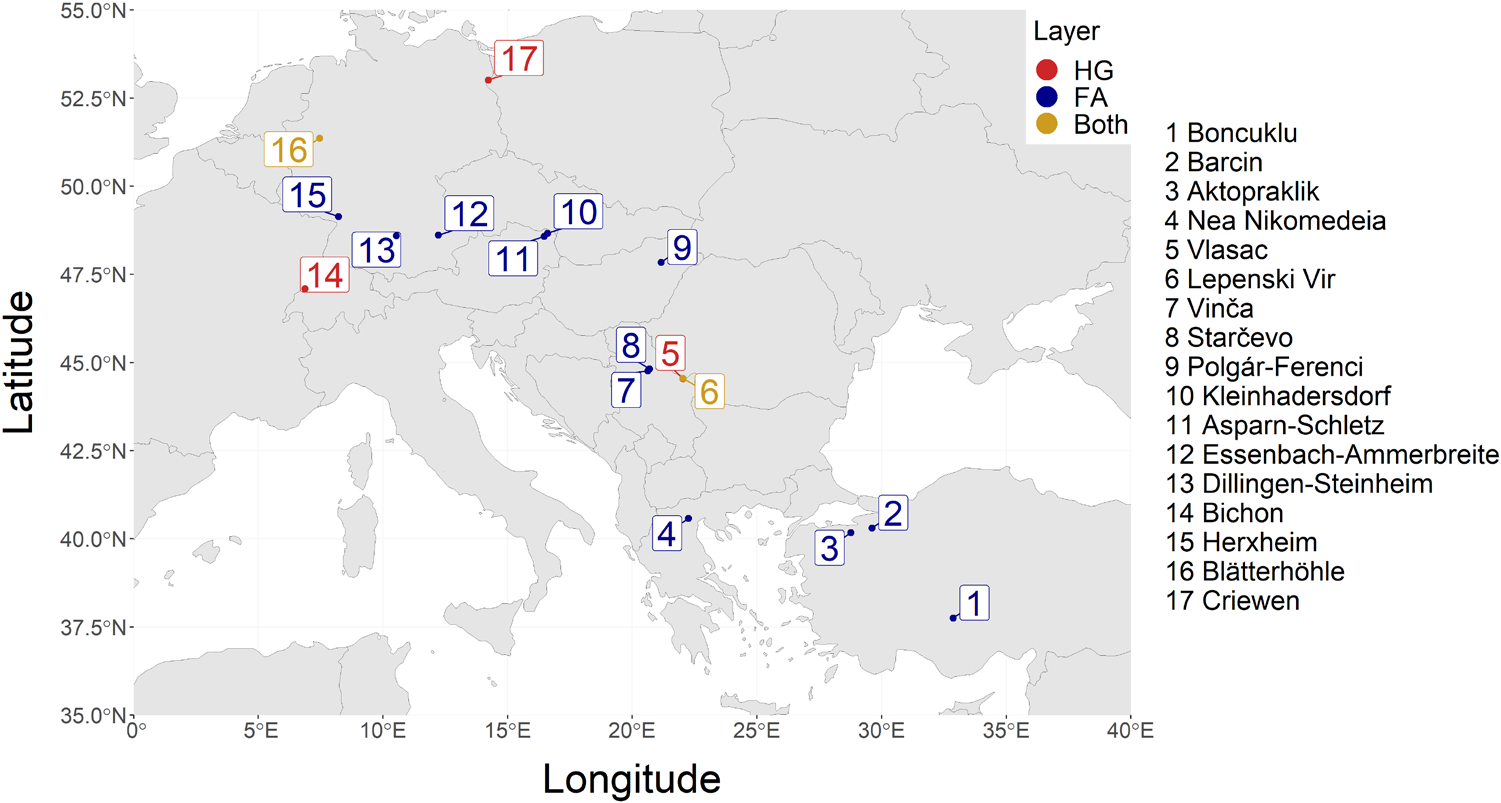
Geographical distribution of the 67 paleogenomes used in the present study. Locations of hunter-gatherer samples are designated with “HG” (red dots) and locations of farmer samples are designated with “FA” (blue dots), while locations in which both hunter-gatherer and farmer samples were used are designated with “Both” (yellow dots).

To investigate the expansion of Neolithic farmers and their interaction with European hunter-gatherers, we analyzed high coverage genomic data from 67 ancient individuals located along a transect between north-western Anatolia and central Europe, from a total of 17 archeological sites (Fig. 4). The majority of the dataset under analysis comprises genomes from Hofmanová *et al*. (*21*), encompassing 49 high-quality target enrichment captures of neutral genomic regions spanning a total of 5 megabases referred to as “neutralomes” (*34*). Per design, they should allow more accurate inferences about demographic events by minimizing bias resulting from functional restrictions or background selection. This dataset was complemented by 18 published genomes of equivalent quality and coverage, fitting the geographic and temporal requirements of our study (data S1). The joint simulation-ABC approach we used requires identical sequencing positions across all genomes. As a result, genomic data generated using different capture libraries or shotgun sequencing could not be included in our analysis. The raw data of the neutralomes and genomes were processed as described in Hofmanová *et al.* (*21*). The dataset consists of 16 Paleolithic-, Mesolithic-, and Neolithic-period hunter-gatherers (HGs) and 51 Neolithic farmers (FAs). Group assignments of each genome as HG or FA were taken from the original publications based on criteria such as the presence or absence of food-producing activities at the site. While the distinction between early Holocene hunter-gatherers and farmers is relatively clear-cut in continental Europe, the picture is more contrasted to the east. For instance, at 11th/10th millennium B.P. Boncuklu, in central Anatolia, small-scale farming was practiced next to hunting and gathering (*67*). At 9th millennium B.P. Lepenski Vir, in the Danube’s Iron Gates, some of the incoming farmers appear to have adopted fishing (*20*). The estimated age of the HG genomes ranges between 13,700 and 5719 yr B.P., while the age of FAs is between 10,078 and 5368 yr B.P. (see data S1 for details).

We used ATLAS [commit = b745621; (*68*)] to infer genotype likelihoods (task = GLF) for all individuals, excluding three bases near the 3′- and 5′-ends of reads. Subsequently, we computed major and minor alleles (task = majorMinor) from these genotype likelihoods, creating a file that delineates variant call sites and their known alleles. As a measure of the genetic differentiation between the genomes, we used the pairwise pseudo-haploid nucleotide diversity. Thus, to get the pseudo-haploid calls, we used the previously generated file with known alleles and ATLAS (task = call, method = majorityBase) to select at each site the base with the most occurrences, filtering out sites with coverage <2×. Then, for each possible pair of genomes, the pairwise nucleotide diversity was estimated with ATLAS (task = estimateF2) as the number of variable sites divided by the number of total sites compared between the two genomes, resulting in 2211 pairwise values, provided in data S2.

We grouped together genomes that were found in the same geographic location, belonging to the same population group (HG or FA) and whose mean ages did not differ by more than 300 years, while maintaining that the maximum intragroup age difference was lower than the minimum intergroup age difference. Therefore, in cases where an individual genome differed by less than 300 years from two groups, it was placed in the group that would guarantee that the intragroup age range would not exceed the intergroup age difference. For example, the samples LepV.HG.2 and LepV.HG.3, which exhibit the smallest such difference, were formed to have a maximum intragroup difference of 122 years and an intergroup difference of 186 years. In total, 23 “population samples” (Table 2) were created for the purpose of creating samples of larger size and reducing the total number of pairwise statistics. We estimated 267 mean values of intrasample and intersample pseudo-haploid nucleotide diversity using the estimated 2211 pairwise values. These 267 mean values of pseudo-haploid nucleotide diversity within and between samples were used as summary statistics in the next steps of the analysis and are provided in data S3.

**Table 2.** Characteristics of population samples used for model choice and parameter estimation. Individuals who were sampled from the same deme and the same generation were grouped together in the simulations to form population samples. The number of samples from each deme, as well as the the size of each sample and their average dates are given in the corresponding columns. In the case of demes with multiple diachronic samples, the values for different samples are separated by comma. The coordinates of each deme are given in WGS84 Plate Carree format (the format used by SPLATCHE3 for the simulations).

Locations in the deme	Population group	Deme latitude (in WGS84 plate carrée format)	Deme longitude (in WGS84 plate carrée format)	Number of samples in the deme	Number of individuals in each sample	Mean age of each sample (in yr B.P.)
Bichon	HG	5243147.8	764764.9	1	1	13,700
Blätterhöhle	HG	5718157	830809	2	1,1	10,602, 5719
Criewen	HG	5901792	1582596	1	2	6635
Lepenski Vir/Vlasac	HG	4958554	2452472	4	2,4,3,2	9630, 8750, 8503, 8069
Aktopraklık	FA	4472220	3202638	2	4,1	8371, 7534
Asparn-Schletz/Kleinhadersdorf	FA	5407677	1833087	2	8,6	7497, 7189
Barcın	FA	4487177	3295858	1	7	8227
Blätterhöhle	FA	5718157	830809	2	2,1	5819, 5416
Boncuklu	FA	4202523	3658504	1	1	10,078
Dillingen-Steinheim	FA	5409760	1173118	1	1	7117
Essenbach-Ammerbreite	FA	5411550	1360050	1	1	6975
Herxheim	FA	5470863.2	914333.8	1	9	6959
Lepenski Vir	FA	4960008	2452011	1	4	7962
Nea Nikomedeia	FA	4517790	2477193	1	2	8141
Polgár-Ferenci	FA	5326244	2354344	1	1	7139
Starčevo/Vinča	FA	4986167	2299304	1	2	7527

### Spatially explicit simulations

To simulate demographic parameters of the Neolithic transition, we used a modified version of the spatially explicit simulator SPLATCHE3 (*69*). SPLATCHE3 uses a map of the studied area in ASCII format, divided into cells, on which are superimposed two layers of simulated populations, each with its own demographic characteristics. Interactions between populations of each layer may occur according to predefined parameters. Each cell thus contains two demes, one per population, HG or FA. Time in the simulations is measured in generations of 25 years, the estimated average human generation interval ~ 6500 yr B.P. (*70*). The simulations consist of two parts, a forward in time demographic simulation and a backward coalescent genetic simulation, which produces DNA sequences for each genome with user-defined times and location, grouped by samples as in the real dataset. We simulated 1600 generations with a starting time of the simulations at ~40,000 yr B.P., corresponding approximately to the onset of modern human migration into Europe (*71*). The mean estimated ages of the observed genomes (data S1) were transformed into the number of generations since the start of the simulation.

The two simulated layers of demes correspond to HGs and FAs, following previous studies (*30*, *33*), and the map used in the simulations is divided into 75 × 47 cells of 100 × 100 km and resembles western Eurasia, including the near East and North Africa (Fig. 1A). Although we focus our analysis on samples distributed along the continental route, we considered a larger area in the simulations to avoid an arbitrary decision on delimiting this route. Coordinates of the observed dataset were transformed into the WGS84 Plate Carree format with the proj4 R package (*72*). The simulations used two zones of HG and FA competition. The area above latitude 43.2° (Fig. 1B) is termed “northwest continental route” hereafter, while the area below that latitude is termed “southeast continental route.” In each cell, admixture occurs unidirectionally at a rate γ from HGs to FAs using the SPLATCHE3 assimilation model (*69*). This means that at each generation, when HGs and FAs coexist in a cell, a fraction γ of the contact between them results in admixture. This leads to the passage of HG genes into the FA population and thus to gene flow from HG to FA, following previous simulation studies (*30*, *33*). Note that the model does not distinguish whether this gene flow is due to the adoption of agriculture by HGs, the assimilation of HGs into the agricultural population, or mixed encounters between individuals from the two populations resulting in the birth of a child in the FA population.

In the modified version of SPLATCHE3 used in this study (available at Zenodo.org), some specific features were added to allow spatiotemporal variation in interactions between the two simulated populations:

1) The admixture rate (γ) is considered to vary both spatially and temporally. Each deme is endowed with a value of γ, which is subject to modification at times specified by the user.

2) Temporally increasing admixture rate. γ can either be the set value for the whole duration of the simulation or start from 0 and increase each generation by a fraction of the set value until it reaches that value. The fraction of γ by which it increases in each generation is referred to as γ_inc_. We applied a model of linear admixture increase for the sake of parsimony, aiming to detect a general trend of variation that differs from a constant rate. Implementing a more realistic model, such as admixture occurring in pulses, would require setting additional parameters, including the number of pulses, their timing, duration, intensity, and spatial distribution.

3) Two competition zones. The competitive interactions between the two simulated layers are modeled using a Lotka-Volterra model of competition (*73*, *74*). Notably, each pair/group of demes within these zones is assigned a unique competition coefficient (α). The utilization of different α for each area allows us to delineate zones where the two simulated populations coexist for varying durations (inversely proportional to the value of α). In all cases, α is considered symmetrical between HGs and FAs in a given zone.

4) Sequencing error. A sequencing error probability (ε) defined by the user has been added to SPLATCHE3, which can now introduce errors to the generated DNA sequences (text S6), to better reproduce observed data.

Parameter values for each simulation were sampled from uniform distributions described in Table 3. The carrying capacity of each deme (parameter *K*) is given as the effective haploid population size. For HGs, the range of possible *K*_HG_ investigated was 270 to 450. The range (density between 0.04 and 0.0675 individuals/km^2^) was based on the 95% confidence interval of the estimated final Paleolithic population in Europe at 13,000 yr B.P. (*75*), multiplying the density by the cell size to get the census population size; dividing it by three to get the reproductive population size of diploid individuals, assuming that only one-third of the population is sexually matured (*76*); and, last, multiplying it by two to get the within deme effective population size in number of gene copies. The range of *K* for FAs (*K*_FA_) was 1200 to 2400 (0.18 to 0.36 individuals/km^2^), starting from the range estimated by Silva *et al.* (*33*) and adjusting it according to preliminary simulations. The population density (*N_t_*) in each deme at each generation *t* is calculated using logistic growth (parameter *r*) and regulated by *K*. The chance of an individual in a deme to migrate at each generation is given as the migration rate (*m*). For HGs, the values of *m*_HG_ and *r*_HG_ are 0.15 and 0.2, respectively, following Currat and Excoffier (*30*). For FAs, *r*_FA_ was set to 0.55, while *m*_FA_ was variable, ranging between 0 and 0.5, which was chosen on the basis of preliminary simulations. The migration can either take place in a stepping-stone manner (*77*) or through LDD, with the fraction of migration events that are LDD being an additional parameter (*P*_LDD_). Even after a HG deme becomes extinct, migrations from neighboring demes and via LDD can still occur toward it. However, these migrations fail to establish lasting populations in cells already occupied by FAs due to competition. The prior range of *P*_LDD_ was set to 0 to 0.025 based on Rio *et al.* (*35*). The distance the migrants travel is drawn from a gamma distribution, defined by a shape (β) and rate (λ) parameter, which are equal to 1.209 and 0.15046, respectively (*35*).

**Table 3.** Model parameter values. For constant parameters, their exact value is provided. For parameters that are varying between simulations, the prior ranges, from which the values are uniformly drawn, are provided.

Parameter	Value
Admixture rate (γ)^*^	0–0.1
Admixture rate in the southeast continental route (γ_S_)^†,‡^	0–0.1
Admixture rate in the northwest continental route (γ_N_) ^†,‡^	0–0.1
Number of generations of γ increase (*t*_inc_)^†,‡^	1–248
Effective population size of HGs (*K*_HG_)^‡^	270–450
Effective population size of FAs (*K*_FA_)^‡^	1200–2400
Proportion of Long-Distance Dispersals (*P*_LDD_)^‡^	0–0.025
LDD shape parameter (β)	1.209
LDD scale parameter (θ)	0.15046
Coefficient of competition in northwest continental route (α_N_)^‡^	0.15–0.37
Migration rate of HGs (*m*_HG_)	0.15
Migration rate of FAs (*m*_FA_)^‡^	0.1–0.5
Growth rate of HGs (*r*_HG_)	0.2
Growth rate of FAs (*r*_FA_)	0.55
Sequencing error rate (ε)^‡^	0.000045–0.00012

*Only used for scenarios AM1 to AM5.

†Only used for scenario AM6.

‡Estimated with AM6.

The competition coefficient between HGs and FAs (α) was initially allowed to take on values in the entire possible range (0.0 to 1.0) and then reduced to 0.15 to 0.37 after a set of exploratory simulations (see text S5) to maximize the number of compatible simulations. Because there were no HGs from the Southern Balkans and western Anatolia included in the dataset, we decided to focus the exploration of the competition coefficient in the Northwest continental route, as defined above, including regions where the HGs cohabitated with the FAs for an extended period of time (*15*). For that purpose, we used a newly implemented feature of SPLATCHE3 that allows for two zones of competition (Fig. 1B), and we set the competition α_N_ in the northwest continental route (defined by latitude larger than 43.2°) to be a variable, while in the rest of the map, α_S_ was set to 1, similarly to previous studies (*33*). In addition, we allowed for different admixture rates in each of those two zones to make this parameter variable in space for scenario AM6. HG individuals can move from the HG layer to the FA layer at a rate γ, which represents the probability that a contact between HGs and FAs will result in gene flow from HGs to FAs, ranging from 0 to 0.1 based on the estimation by Silva *et al.* (*33*) and adjusted according to preliminary simulations. The sequencing error rate (ε) was set between 0.000045 and 0.00012, with a range chosen from preliminary simulations.

For each genome, we simulated 50 independent loci of 1000 base pairs each, without recombination, using a mutation rate of 2.15 × 10^−8^ mutations per nucleotide site per generation based on the estimations of Nachman and Crowell (*78*). As demonstrated by text S7, we had to strike a balance between the high computation time required to simulate each locus and the accuracy of the statistics. We therefore decided to use the empirical average of pseudo-haploid nucleotide diversity over the 5000 loci and compare it with the average calculated over a much smaller, but sufficient, number of simulated loci (see text S7). In the modified version of SPLATCHE3 used, the pseudo-haploid nucleotide diversity within and between samples is calculated directly by the program and written in a separate output file with the extension “.*PseudoHaploDiv*.”

#### Six investigated scenarios with different admixture modes

To investigate whether the rate of admixture between HGs and FAs was uniform along the continental route and whether it became more frequent the longer their cohabitation went on, we started by elaborating six scenarios, which differed in the spatial and/or temporal mode of admixture (Fig. 1D). These six scenarios (named AM1 to AM6) were inspired from those described in a previous study from our group (*33*):

1) AM1. Constant admixture rate in time and space: The parameter γ remains constant across the entire geographical map, maintaining its values from the initial interaction between HGs and FAs to the conclusion of the simulations. This scenario mirrors the demic diffusion model, where a limited number of HGs integrate into the farming community in each deme at a uniform rate over space and time (*30*).

2) AM2. Spatially increasing admixture rate: In this scenario, the parameter γ remains constant over time but exhibits a gradual increase as one moves from Anatolia toward central Europe. This scenario reflects a variant of the demic diffusion model, including a gradient of admixture between HGs and FAs with higher rates of admixture toward the northwest regions of the continental route.

3) AM3. Temporally increasing admixture rate: In this scenario, the parameter γ is initially equal to 0 across all demes. However, upon colonization by FAs, γ within a deme progressively increases with each succeeding generation at a rate denoted as γ_inc_, until it reaches its maximum value equal to γ at the end of the cohabitation time with HG. γ is constant across all demes. This scenario represents a variant of the demic diffusion model incorporating an escalating admixture rate over time following the introduction of farming into a particular cell (region).

4) AM4. Spatially and temporally increasing admixture rate: In this combined scenario, the parameter γ exhibits a dual pattern of increase; it escalates with distance from Anatolia across geographical space, and it also increases with each successive generation as HGs and FAs coexist within demes. This scenario integrates features from both AM2 (spatial gradient) and AM3 (temporal escalation) reflecting a nuanced demic diffusion model. In this scenario, the admixture rate reaches its maximum value at the end of the cohabitation time between HGs and FAs.

5) AM5. Spatially decreasing admixture rate: In this scenario, the parameter γ remains constant over time but decreases as one moves from Anatolia to central Europe. Unlike AM2, where there is a spatial gradient of increasing admixture, this scenario represents the opposite trend, with admixture rates decreasing toward the northern regions along the route from Antolia to central Europe.

6) AM6. Variable duration of admixture rate increase: In this scenario, the parameter γ increases with each generation for a variable period, regulated by the parameter *t*_inc_, as long as hunter-gatherers (HGs) and farmers (FAs) coexist within the same cells. The admixture rate reaches its maximum after a variable number of generations *t*_inc_, representing a proportion of the local cohabitation period. In addition, the model includes two spatial zones of admixture, with γ being uniform within each zone but potentially different between them. As a result, the admixture rate may be spatially constant along the continental route or vary, either increasing or decreasing toward the northwest, and may also remain stable or increase locally over time.

Note that a scenario involving temporally decreasing admixture rates was not explored, as it has been previously dismissed by Lipson *et al*. (*19*).

For scenarios with spatially varying admixture rates (AM2, AM4, and AM5), the region between south-west Asia and central Europe was subdivided into 10 zones. Each of these zones was assigned a value for the parameter γ that differs (positively or negatively) from the rate in the previous zone by 10% of the maximum γ. In AM6, the spatial aspect of admixture was integrated by exploiting the same concept of defining two zones as with differing competition, similar to the southeast continental route and northwest continental route described earlier. This setup allowed us to test whether one zone exhibited higher values of γ compared to the other (indicating spatially increasing or decreasing γ) or if both zones maintained the same γ values (indicating that γ could be considered constant in space).

To determine the speed γ_inc_ at which γ needs to increase in each generation for scenarios AM3, AM4, and AM6, we conducted 200,000 preliminary simulations. These simulations were based on the parameter priors outlined in Table 3 and used scenario AM1 as the starting point. From those preliminary simulations, we retrieved the mean cohabitation time between HGs and FAs and used the mgcv R package (*79*) to fit a generalized additive model (GAM), where the mean cohabitation time was regressed against *K*_HG_, *K*_FA_ and α_N_. For each simulation of scenarios AM3, AM4, and AM6, the GAM was used, with the three aforementioned parameters as input, to predict the mean cohabitation time. For scenarios AM3 and AM4, the value of γ_inc_ in each simulation was set to the inverse of the predicted mean cohabitation time, so γ would reach its maximum value by the end of the cohabitation period. For AM6, to investigate the temporal heterogeneity of γ, we randomly draw for each simulation a number of generations *t*_inc_, ranging between 1 and the mean cohabitation time of HGs and FAs, and we set the value of γ_inc_ to the inverse of the chosen number of generations *t*_inc_. That way, γ could be constant in time (if the chosen number of generations was 1) or increase for a flexible time period, up to the maximum mean number of generations of cohabitation, potentially allowing the estimation of the duration of the period over which the admixture rate increased. The number of generations during which the γ increased ranged from 1 to 248. However, it did not follow a uniform distribution, as its values depended on the estimated mean cohabitation time for each simulation. Therefore, it is important to stress here the difference in temporal γ increase between scenarios: While γ was increasing steadily with each passing generation until reaching its maximum value at the end of the cohabitation period in scenarios AM3 and AM4, in scenario AM6, it was set to increase for a specific number of generations and then retain its maximum value until the end of the cohabitation period (see Fig. 1D).

We performed 800,000 simulations per scenario (AM1 to AM6), of which 33,500 simulations per scenario were retained for further analysis, for a total of 201,000 simulations. This subset refers to simulations that were able to reproduce the observed dataset, meaning those that produced all the observed samples at the appropriate generation, location, and population layer (HG, Fig. 1B or FA, Fig. 1C), and for which the HGs went extinct before the end of the simulation. We used a two-step ABC procedure. In the first step, we performed a straightforward ABC rejection sampling based on the spatiotemporal characteristics of the samples, a process we refer to as the “demographic filter.” Simulations, where not all genomes could be sampled (usually because HGs went extinct too early or where HGs survived later than what is assumed), were not retained for the second step. The posterior distribution from the first step was subsequently used as an informed prior for the second step, which involved model selection based on molecular data. The number of simulations that passed the demographic filter for the various scenarios ranged between 33,500 and 36,000 depending on the scenario. Therefore, if more than 33,500 simulations successfully passed the demographic filter for any given scenario, then a subset of 33,500 simulations was drawn randomly to get an equal number of simulations for the next step, the model choice procedure. To evaluate the robustness of the scenario comparison, we built a confusion matrix by using an ABC-random forest approach using all retained simulations for each scenario. Because five of the scenarios (AM1 to AM5) were not well differentiated in the confusion matrix, they were tested by using a simulated dataset with samples distributed homogeneously in space and time to verify their ability to produce different/meaningful results. This testing relied on diachronic samples collected from evenly spaced locations along the Danube route (see text S1). In addition, although scenario AM6 is the easiest to distinguish, it shares characteristics with all the other scenarios, so we performed another model choice using only the five other scenarios (AM1 to AM5). The goal was to get a more detailed comparison between scenarios with defined admixture characteristics (text S3). Lastly, we tested the effect of the level of γ on the misclassification of the simulations generated under these five scenarios. This was done by dividing the simulations in two groups based on their γ value, performing the ABC analysis for each group and contrasting the results of the two simulation groups. These results are presented in text S8.

#### Estimation of parameters for the continental Neolithic expansion

Next, we made demographic inferences regarding the human populations along the continental route to better understand the Neolithic expansion in Europe by estimating parameters regarding the demography of the involved populations (HGs and FAs), the migration of FAs, and their admixture with HGs. For the parameter estimation, we used scenario AM6, as it was identified as the most probable demographic scenario among the six investigated during the ABC model choice. For AM6, we performed additional simulations, for a total of 2,400,000, 100,000 of which were retained after applying the demographic filter.

The average distance of the LDD events in our simulations was 800 km. To test if shorter LDD events affect the resulting levels of pseudo-haploid nucleotide diversity, we performed a set of simulations using scenario AM6 and an average LDD distance of 300 km. In addition, we performed a set of simulations, where all FA migrations were short scale (*P*_LDD_ = 0), to investigate the importance of LDDs in replicating the observed dataset.

Because the simulation framework we used does not include geomorphological characteristics, such as mountain ranges, the migration of modern humans into Europe can happen either through Anatolia and the Balkans, or through Caucasus and the Eastern European plains. To confirm that migration into Europe through these two routes simultaneously does not produce different results compared to migration only through Anatolia and the Balkans, we performed a set of simulations under scenario AM6 that additionally included a barrier in Caucasus that prevented migration through this area. This investigation is presented in text S9.

### Statistical analysis: ABC model choice and parameter estimation

The choice of the most likely scenario was performed with the abcrf R package (*80*), which uses a random forest approach to perform the ABC selection. The choice of abcrf was motivated by its superior discriminative power with a lower number of simulations compared to other ABC methods (*80*). Furthermore, abcrf is capable of handling a large number of summary statistics effectively. In our study, we used 267 summary statistics of mean pseudo-haploid nucleotide diversity within and between samples, leveraging the robust capabilities of abcrf for our analyses (see text S1). The random forest ABC analysis outputs the number of trees that selected each model and the posterior probability of the best model. The forest consisted of 2000 trees after checking that a larger number of trees does not considerably decrease the scenario misclassification rate.

Given that abcrf does not formally evaluate the fit of the simulated data to the observed data, we assessed the probability of each of the five scenarios reproducing the observed data by using ABCtoolbox2 (*81*). This assessment involved calculating the marginal density *P* value, where the null hypothesis posits that the simulated data fit the observed values adequately. Because of the high number of summary statistics, we used the first 10 components that best explain the variability of the data for estimating the marginal density *P* value. The components resulted from the computation of linear combinations of the 267 values of intra- and intersample pseudo-haploid nucleotide diversity using partial least squares [PLS; (*82*)]. This transformation was done with the “find_pls.R” R script and the “transformer” software (both included in ABCtoolbox2) to reduce the dimensionality of the dataset. The number of components was chosen on the basis of the root mean square error (RMSE; fig. S15), which was also estimated with the find_pls.R R script. For all estimated parameters, using more than 10 components reduces the RMSE by less than 2%, and therefore, only 10 components were used for the transformation. The marginal density is estimated on retained simulations based on a tolerance level (δ) and the Euclidean distance between each simulated summary statistic and the observed ones. For scenarios AM1 to AM6, during the model choice, the parameter δ was fixed at 0.03, and we retained 1000 simulations per scenario. Two additional values of δ were tested, and the results are presented in table S1. During the parameter estimation, we reassessed the fit of scenario AM6 by using three values of the parameter δ (0.005, 0.01, and 0.05), with 500, 1000, and 5000 simulations retained, respectively.

We made demographic inferences by estimating the most probable values of the parameters used in the simulations with scenario AM6 (‡ in Table 3). The parameter estimation was performed with ABCtoolbox2, which calculates the minimized Euclidean distance between simulated and observed statistics. Based on these distances, ABCtoolbox2 retains a fraction of the simulations that are closer to the observed data and computes the posterior distribution of the parameter values (*81*). This is done using an ABC-GLM regression adjustment (*83*). We performed the parameter estimation using three values of δ, 0.005, 0.01, and 0.05, retaining 500, 1000, and 5000 simulations, respectively. The results for δ = 0.01 are presented in the main text, while the results of the other values of δ are presented in table S8. For the two γ variables (γ_S_ and γ_N_) and the two *K* variables (*K*_HG_ and *K*_FA_), we estimated two-dimensional posteriors and used a Markov chain Monte Carlo to sample from these posteriors, to investigate if there are combinations of these variables that are more probable than others.

The summary statistics used for the parameter estimation were the first 10 components resulting from the PLS transformation of the pseudo-haploid nucleotide diversity. For each parameter, we estimated the posterior range, as the 95% HDI of the distribution, and we reported its mode and mean. We evaluated the precision of our point estimates by calculating their relative bias = $\frac{1}{n}\sum_{i=1}^{n}\frac{|\mu_{i}-\chi_{i}|}{\chi_{i}}$ , where *n* is the number of pseudo-observations, χ*_*i*_* is the pseudo-observed parameter value, and μ*_i_* is the mean or mode of the posterior parameter distribution of the replicate. To do this, we considered the summary statistics of each retained simulation, in turn, as pseudo-observed statistics with known parameter values, and we performed the parameter estimation procedure with all other simulations.

## Supplementary Materials

### The PDF file includes:

Texts S1 to S9 Figs. S1 to S15 Tables S1 to S9 Legends for data S1 to S3
