# Supplementary material for: Local increases in admixture with hunter-gatherers followed the initial expansion of Neolithic farmers across continental Europe
Source: Sci Adv. 2025 Aug 20;11(34):eadq9976. doi: 10.1126/sciadv.adq9976 (PMC12366693; doi:10.1126/sciadv.adq9976)
Supplement: Supplementary file 1 — Texts S1 to S9 Figs. S1 to S15 Tables S1 to S9 Legends for data S1 to S3 [file sciadv.adq9976_sm.pdf]

Science Advances
AAAS

# Supplementary Materials for

## Local increases in admixture with hunter-gatherers followed the initial expansion of Neolithic farmers across continental Europe

Alexandros Tsoupas *et al.*

Corresponding author: Mathias Currat, mathias.currat@unige.ch

*Sci. Adv.* **11**, eadq9976 (2025)
DOI: 10.1126/sciadv.adq9976

**The PDF file includes:**

Texts S1 to S9
Figs. S1 to S15
Tables S1 to S9
Legends for data S1 to S3

**Other Supplementary Material for this manuscript includes the following:**

Data S1 to S3

**Text S1**
<u>Theoretical investigation of five scenarios</u>

*Objective*: In the model choice cross-validation performed in the main text analysis, five (AM1-AM5) of the six investigated scenarios had a high misclassification rate (Fig. 2). In order to check whether the scenario classification is confounded by the heterogeneous spatiotemporal sampling of the observed dataset and whether the random forest ABC (*80*) has enough discriminatory power, even at low numbers of simulations, we performed a set of exploratory simulations with SPLATCHE3. We excluded scenario AM6 from this analysis, as it was clearly distinct from the others.

The five investigated scenarios (AM1-AM5) are the same as in the main text, see Material and Methods for details about the parameters. The only difference is the molecular dataset we used. Here we used an "ideal" dataset, homogeneous in space and time, to remove the possibly confounding effect of heterogeneous sampling on scenario classification. The sampled dataset consisted of both HGs and FAs in five locations and at five different times for each location, for a total of 50 samples. The sampling locations were in equidistant demes (fig. S1). The first sampling date was shortly after the colonization of the entire map by FAs (generation 1,250), and we sampled in increments of 30 generations (~750 years). The parameter priors were the same as in Table 3 of the main text.

We performed 25,000 simulations per scenario. 13,000 simulations were retained from each scenario, after filtering out the ones missing samples (demographic filter, see main text). Contrary to the main text, we retained the ones where the HGs existed until the end of the simulation, since our goal was a theoretical investigation of the scenarios and not to replicate the observed demographic history of the Neolithic.

We constructed a confusion matrix with abcrf (*80*), using as summary statistics all the pairwise values of inter- and intra-sample pseudo-haploid nucleotide diversity (fig. S2), as in the main text analysis.

The model choice validation with abcrf using an artificial dataset homogeneous in space and time shows variation in the accuracy of scenario identification when using all the pairwise values of pseudo-haploid nucleotide diversity as summary statistics. The misclassification rate is 0.55, with scenarios AM1 and AM2 having a probability of ~0.55 to be correctly identified, which means the constant admixture rate and the spatially increasing one create an identifiable pattern of nucleotide diversity. The two scenarios with temporally increasing admixture rate (AM3 and AM4) were hardly distinguishable from one another, but together they were clearly differentiated from the other three scenarios of temporally constant admixture rate (AM1, AM2 and AM5), which means that the temporal increase of the admixture rate creates a distinct pattern of nucleotide diversity but also masks the spatial pattern. Despite the relatively high misclassification rate, there exists a detectable signal that differentiates the simulated scenarios with the information lost resulting from the pseudo-haploidization of the genomic data. Therefore, it appears that the spatial and temporal heterogeneity of the observed samples is a factor that contributes to the misidentification of scenarios, but it is not the sole one, as even with an artificially homogeneous dataset the scenario misclassification rate is relatively high. Additionally, we tested if using a PLS transformation on the data (see main text), improves the ability of abcrf to correctly identify the scenarios. We transformed the pairwise values of inter- and intra-sample pseudo-haploid nucleotide diversity into PLS components for all five scenarios and we used the first ten components and abcrf to construct a confusion matrix (fig. S3). It shows

that a PLS transformation does not improve the ability of ABC to identify the various scenarios. For this reason, we chose to use the original pairwise values of inter- and intra-sample pseudo-haploid nucleotide diversity as summary statistics for the scenario choice on the observed data.

**Text S2**
Model choice and parameter estimation omitting genomes from Lepenski Vir

*Objective*: The categorization of genomes from Lepenski Vir as hunter-gatherers (HG) or farmers (FA) is challenging (*20*). To remove the uncertainty of the classification from our analysis, we performed the model choice and the parameter estimation after removing the Lepenski Vir samples from both the observed and the simulated summary statistics. Of the original 67 genomes, 59 were retained for this analysis.

*S2.1 Model choice using scenarios AM1-AM6*

All six investigated scenarios were able to reproduce the observed summary statistics, after removing the Lepenski Vir genomes (table S3).
The model choice showed that scenario AM6 was still the most probable (table S4), with its posterior probability (0.7) being very close to the one estimated when including the Lepenski Vir genomes (0.77).

The confusion matrix (fig. S4) is very similar to the one based on summary statistics that included Lepenski Vir genomes (Fig. 2 of the main text), which shows that the samples from Lepenski Vir did not confound the scenario differentiation.

*S2.2 Parameter estimation using scenario AM6*

As in the main text, the parameter estimation was performed using scenario AM6. The marginal posterior density p-value of the scenario was 0.11, given a tolerance of 0.01. The characteristics of the posterior distributions of most parameters (table S5 and fig. S5) are quite similar to the estimations using the Lepenski Vir genomes. The only important difference is found in the mode of the posterior distribution of the generations of admixture rate increase ($t_{inc}$), as the mode without the Lepenski Vir (36) is approximately two times lower than the mode with Lepenski Vir (70). However, both estimations (with and without Lepenski Vir) are characterized by closer means (72 and 62 respectively), large relative posterior biases and overlapping 95% HDI. Therefore, we cannot safely conclude that the number of generations of admixture rate increase estimated with and without the genomes from Lepenski Vir are actually different.

The above analyses show that the uncertainty in group classification of the genomes from Lepenski Vir does not substantially affect the results and the estimations performed in the main text.

**Text S3**

Model choice using five of the investigated scenarios

*Objective*: The comparison of all six scenarios showed that the scenario that best describes the observed data is AM6 (see main text), in which the admixture rate increases during a variable period of time and can also vary spatially, between two geographical zones. Parameter estimation based on this scenario indicated that the admixture rate increased over approximately 70 generations (Table 1, main text), but provided no information on whether the rate varied spatially across geographical regions. Since AM6 shares features with all other scenarios yet is clearly distinguishable from them, we excluded it and repeated the model choice to examine the relationships among scenarios AM1-AM5 in greater detail.

The confusion matrix (fig. S6) displays the same image as the one including all 6 scenarios (Fig. 2), namely that scenarios with temporally increasing admixture (AM3 and AM4) are distinct from scenarios with temporally constant admixture (AM1 and AM2). However, scenarios with the same temporal but different spatial model of the admixture rate cannot be clearly differentiated from one another (AM1 from AM2 and AM3 from AM4). This means that while our dataset can inform on the temporal dynamics of the admixture rate, it provides limited insight into its spatial pattern.

The model choice shows that the most probable scenario is AM3, the scenario with temporally increasing admixture rate, with a posterior probability of 0.43 (table S6). Thus, model choice based on the subset of five scenarios (AM1–AM5) corroborates the inference drawn from the best-supported scenario, AM6 (Fig. 3, main text). It supports an increasing admixture rate over time along the Continental route, although we cannot definitively rule out a simultaneous spatial increase in a north-western direction, as scenarios AM3 and AM4 remain difficult to distinguish.

**Text S4**

Model choice using three of the investigated scenarios

*Objective*: Since the comparison of the five scenarios (Supplementary Text 3) showed that both scenarios with temporally increasing admixture rate (AM3 and AM4) are hardly distinguishable, while the scenario with admixture rate decreasing with space (AM5) is almost unidentifiable, we decided to perform again the model choice using only three of the scenarios to avoid noise: AM1 as reference with admixture rate constant in time and space, AM2 for investigating a spatial pattern in the admixture rate and AM3 for investigating a temporal pattern. The model choice shows again that the most probable scenario is the one with temporally increasing admixture rate (table S7).

The confusion matrix (fig. S7) shows that, while the admixture rate increasing with time produces different results than when it is constant, the spatial heterogeneity of admixture rate can hardly be distinguished from a scenario of the admixture rate being constant in space.

**Text S5**

Exploration of the range of competition coefficient

*Objective*: We started by exploring the whole range of possible values of the competition coefficient $\alpha_N$ in the Lotka-Volterra model of competition between HGs and FAs along the Continental route of the Neolithic Transition. The goal was to reduce the prior distribution in order to optimize computational efficiency.

We performed 25,000 simulations using the modified version of SPLATCHE3 (*69*) described in the main text, allowing for a variable coefficient of competition $\alpha_N$. We followed the same framework as for the simulations of scenario AM6 in the main text. The parameter priors were the same as in Table 3 of the main text, except for $\alpha_N$, for which we explored the whole range of possible values, between 0 and 1. 151 of the simulations (0.6%) passed the demographic filter by successfully reproducing the observed dataset. The posterior range of the competition coefficient is 0.13-0.39, with a 95% Highest Density Interval (95% HDI) of 0.15-0.37. The distribution of values can be seen in fig. S8.

Our results show that very low or high values of competition coefficient lead to simulations unable to produce the observed dataset. Therefore, in the following simulations, we reduced the prior range of $\alpha_N$ to 0.15-0.37 to optimize the computational time by avoiding simulating unsuccessful simulations (i.e., not allowing to produce all samples).

**Text S6**
<u>Implementation of reads sequencing error in SPLATCHE3</u>

*Objective*: SPLATCHE3 does not produce sequencing reads but full DNA sequences without errors, so a fixed error rate was implemented to mimic read errors for pseudo-haploid genomic data as follows:

Table S9 gives the probability to find no difference $P_{nodiff}$ between two genomes (whether intra or inter population samples) at the pseudo-haploid level using the majority allele, given a sequencing error rate ε, which will be set as parameter for SPLATCHE3, and assuming a depth >2.

If both genotypes are identical (00x00 or 11x11), then one allele is taken at random for each pseudo haploid genome. The probability that they do not differ is either that none has a sequencing error, with a probability $(1-\varepsilon)*(1-\varepsilon)=(1-\varepsilon)^2$ , or both have a sequencing error with a probability equal to $\varepsilon*\varepsilon=\varepsilon^2$. If only one allele has a sequencing error, then both pseudo-haploid genomes will be identified as different.

If both genotypes are homozygous but for a different allele (00x11 or 11x00), then one allele is taken at random for each pseudo haploid genome. The probability that they do not differ results from one of the two alleles having a sequencing error but not the other, with a probability equal $\varepsilon(1-\varepsilon)$, or the reverse, leading to $2\varepsilon(1-\varepsilon)$. If both alleles have a sequencing error, or both have no sequencing error, they will be identified as different.

If at least one genome is heterozygous, (01x11 or 01x00 or 01x01 or 00x01 or 11x01), then the probability that the two pseudo haploid genomes do not differ is always equal to ½ because one allele will always be 0 (or 1) and the other will have a probability ½ to be 0 or 1.

The implementation in SPLATCHE thus consists in counting the number of differences between each pair of simulated diploid genomes by looping over all positions and 1) draw a random number RN between 0 and 1, 2) compare RN to the corresponding $P_{nodiff}$ from the above table depending on both genotypes; 3) if RN > $P_{nodiff}$ add one nucleotide difference between the two genomes. At the end of the loop the number of differences is divided by the total number of compared positions.

Note that this equation is not taking into account post-mortem damage (PMD), which would increase the error rate a little. However, as the goal is not to estimate this error rate but to take it into consideration in the analyses by using a prior distribution, it should not bias the results as the effect of PMD would be included in the prior range.

**Text S7**

Investigation of the effect of the number of simulated genetic loci on simulated pseudo-haploid nucleotide diversity and computational time

*Objective*: In our study, the observed genetic data that were used consisted of approximately 5,000 neutral independent genetic loci of 1000 bp each (*34*). However, as our analyses are based on spatially explicit simulations, simulating such a high number of genetic loci is not feasible, especially as we want to explore a large number of combinations of model parameters. A balance must be struck between the number of simulated loci and the computational time. For this reason, we investigated how the number of simulated genetic loci affects the computational time and the levels of simulated pseudo-haploid nucleotide diversity.

We performed simulations under scenario AM6, using the same parameter priors as for the simulations of the main text (see Table 3). For each number of loci between one and 480, we performed 100 simulations, for a total of 48,000 simulations. For each simulation, we recorded the completion time, measured in seconds (s), and calculated all 267 inter- and intra-sample pseudo-haploid nucleotide diversity values. These values, which were also estimated for the observed data, served as summary statistics in the ABC analysis described in the main text.

As expected, the simulation time shows a positive linear relation with the number of simulated genetic loci (fig. S9). Then we averaged the 267 values pseudo-haploid nucleotide diversity for each simulation and estimated their standard deviation (SD) to evaluate how they vary depending on the number of simulated loci. The per simulation mean pseudo-haploid nucleotide diversity and the SD were plotted against the number of simulated loci (fig. S10 and fig. S11 respectively). From figs. S9-S11, we saw that by simulating 50 loci we can keep the simulation duration of a single simulation under 30 seconds, while greatly reducing the variance of the mean and the standard deviation of the summary statistics.

Lastly, we checked the difference between each summary statistic's mean obtained after a large number of simulations under scenario AM6 (38,000) with either 50 or 150 loci. We computed the relative bias for each of the 267 summary statistics ($s$) as:

relative bias = $\sum_{i=1}^{n} \frac{|\bar{s}_{150i} - \bar{s}_{50i}|}{\bar{s}_{150i}} \times 100/n$, where $n$ is the number of statistics, $\bar{s}_{150i}$ is the mean of each statistic from simulations of 150 genetic loci, and $\bar{s}_{50i}$ is the mean of each statistic from simulations of 50 genetic loci In this way we got a relative estimate of how much the simulated statistics differed when using three times more simulated loci. According to this estimation, the statistics resulting from 50 loci ($s_{50}$) differed on average by 0.06% from the statistics resulting from 150 loci ($s_{150}$), with the maximum difference being 0.21%.

Thus, simulating 50 loci allows for a significant reduction in computational time while maintaining, on average, the same levels of pseudo-haploid nucleotide diversity. Consequently, 50 loci were selected as the number of simulated loci for the main analyses in our study, allowing us to efficiently explore the parameter space.

**Text S8**

Identifying the effect of admixture rate ($\gamma$) level on scenario classification

*Objective*: Since the prior distribution of admixture rate ($\gamma$) values for our simulations ranged between 0 and 0.1, there is the possibility that simulations with low values of $\gamma$ produce similar levels of pseudo-haploid nucleotide diversity between five of the investigated scenarios (AM1-AM5), increasing the model misclassification rate. To test this, we performed the ABC model choice cross-validation on two different sets of simulations, as follows.

We retained only scenarios AM1-AM5, which had high misclassification rates in the confusion matrix (Fig. 2). We divided the simulations that were performed for the model choice in the main text into two groups. One group included the simulations with admixture rate values that fell within the lower half of the $\gamma$ prior range (0-0.05) and the other group included the simulations with $\gamma$ values that fell within the upper half of the admixture rate prior range (0.05-0.1). We then used the two groups of simulations to perform the model choice twice with the abcrf R package (*80*), using the pairwise values of inter- and intra-sample pseudo-haploid nucleotide diversity as summary statistics.

According to the confusion matrices (fig. S12), the misclassification rate decreases for the higher values of $\gamma$, which confirms that lower $\gamma$ values create less distinguishable patterns of molecular diversity among scenarios. Additionally, scenarios AM3 and AM4, the two scenarios with temporally increasing $\gamma$, are less probable to be misclassified as scenarios with temporally constant $\gamma$ for higher values of $\gamma$, although it is still quite probable to be misclassified as one another. The same occurs for scenarios AM1 and AM2, which for higher values of $\gamma$ are less probable to be misclassified as scenarios with temporally increasing $\gamma$, but are still misclassified as one another. These indicate that with higher values of $\gamma$, it is easier to differentiate the temporally increasing from the temporally constant $\gamma$, but the spatially constant admixture rate is still difficult to be differentiated from the spatially increasing one.

We also performed model selection using each of the two groups of simulations, both of which identified AM3 as the best model, with a probability of 0.40 for the lower half of the $\gamma$ prior range and 0.45 for the upper half. In the parameter estimation conducted using scenario AM6 (see main text), the mean estimated $\gamma$ ranged from approximately 4.6% to 4.9%, with the mode falling between 2.4% and 2.9%. This indicates that the most probable values were within the lower half of the prior range, which partly explains the challenges we faced in distinguishing among these five scenarios during model selection.

**Text S9**

Exploration of a geographical barrier in Caucasus

*Objective*: Our simulation framework did not account for geomorphological barriers that might restrict population movements, as we applied uniform demographic (*K* and *r*) and migratory (*m* and *LDD*) parameters across the landscape. Consequently, during their simulated expansion, FAs could enter Europe either via Anatolia through the Balkans or by moving north through the Caucasus and subsequently entering Europe through the Pontic steppes. Given the spatially explicit nature of our approach and the positioning of our samples along the route from Anatolia to Central Europe, the Balkan route offers a significantly shorter pathway from the source of the simulated farmer expansion compared to the Caucasus route, which would require approximately 40 additional generations. To ensure the robustness of our results, here we evaluated whether enabling migrant passage through the Caucasus in our simulations had any impact. Specifically, we sought to determine whether the introduction of an explicit geographical barrier in the Caucasus would alter the pseudo-haploid nucleotide diversity levels observed in the investigated populations, compared to a scenario without such a barrier.

We performed 17,000 simulations for each of two scenarios, using the same framework and parameter priors as for scenario AM6 of the main text. In the first scenario (No Barrier) we used the same simulated map as for scenario AM6, in which FAs can cross the Caucasus. In the second scenario (Barrier at Caucasus), a barrier was added in Caucasus preventing the northward movement of populations through this route (fig. S13). For the two scenarios we plotted the distribution of the resulting simulated values of pseudo-haploid nucleotide diversity. The presence of a barrier in Caucasus did not affect the distribution of the pseudo-haploid nucleotide diversity values as seen in fig. S14.

Additionally, we performed an ABC cross-validation model choice between the two scenarios, with the abcrf R package (*80*), and using the observed values of pseudo-haploid nucleotide diversity as summary statistics. The ABC model choice identified the scenario including a barrier in Caucasus as the most probable, with a posterior probability of 0.52. This low probability means that the scenario without a barrier is almost equally probable and indicates that the presence of the barrier does not affect the resulting pattern of pseudo-haploid nucleotide diversity in a way distinguishable between the two scenarios. This was further reinforced by the confusion matrix of the two scenarios. Each of them had only 0.49 probability to be correctly identified, which means that the two scenarios do not produce sufficiently differentiated data to be identified. Due to the above analysis, for the simulations of the main text we retained the simplest of the two scenarios for the main text, the one without a geographical barrier in Caucasus.

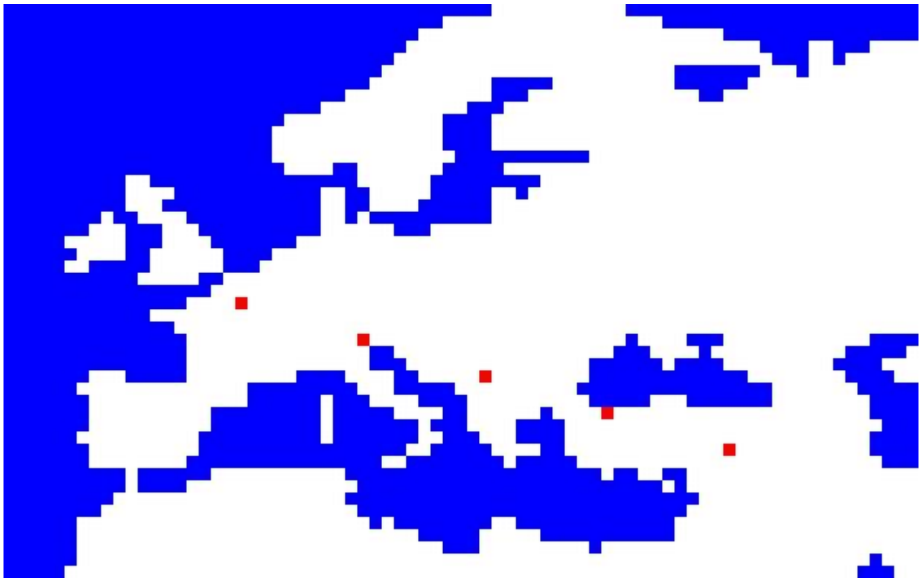

**Fig. S1. Sampling locations for the theoretical exploration of scenarios.** This map was used in the simulations investigating the differences between scenarios AM1-AM5. From each location marked in red, five diachronic samples of hunter-gatherers and five diachronic samples of farmers were drawn.

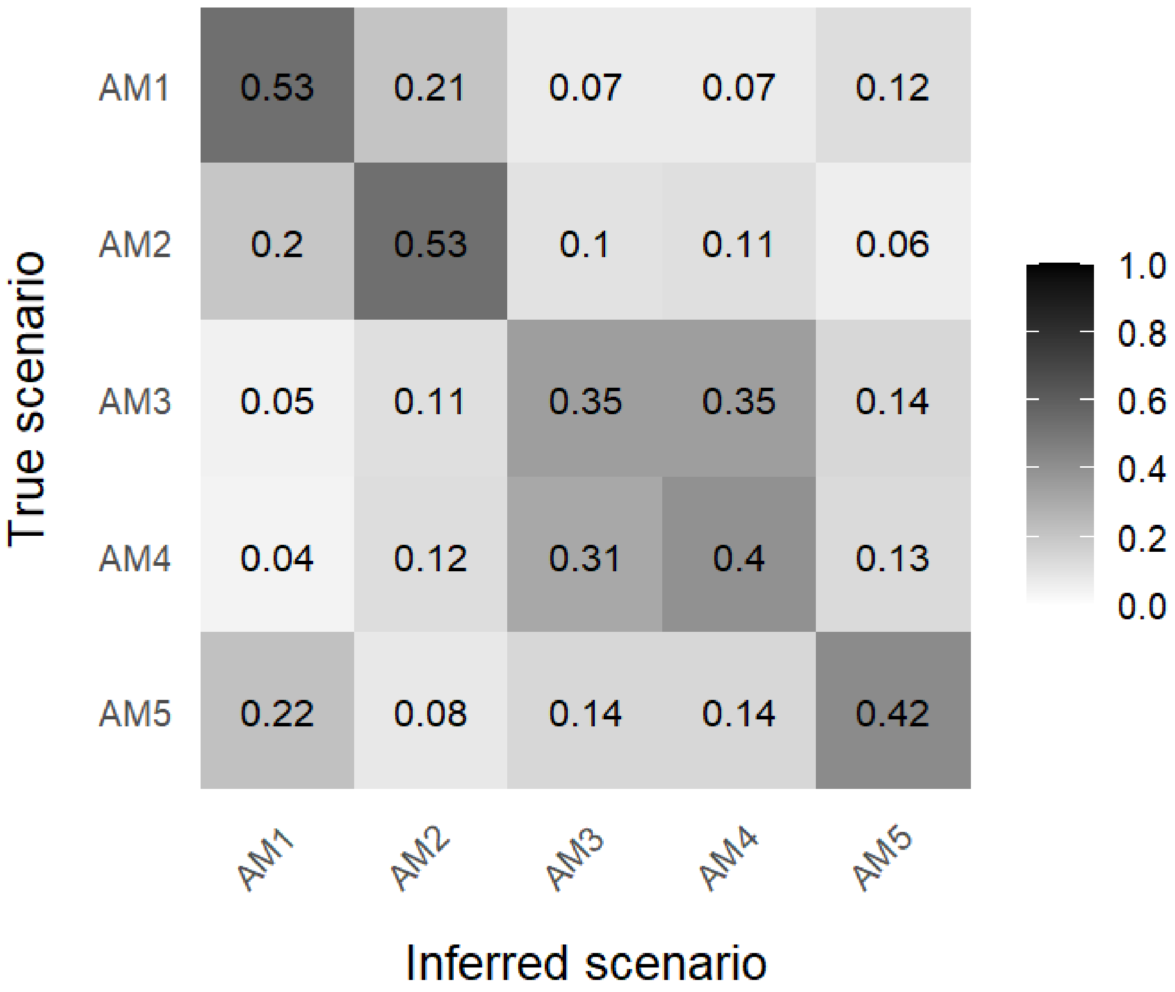


**Fig. S2. Confusion matrix of the theoretical investigation of five of the investigated scenarios (AM1-AM5) using pseudo-haploid nucleotide diversity.** Calculation performed with abcrf R package (ABC-random forest approach, *80*), using 13,000 simulations per scenario and 2,000 trees. Each row represents simulations from a given scenario (the 'true' scenario), while the columns indicate the proportion of those simulations that ABC attributed to each of the studied scenarios (the 'inferred' scenarios).

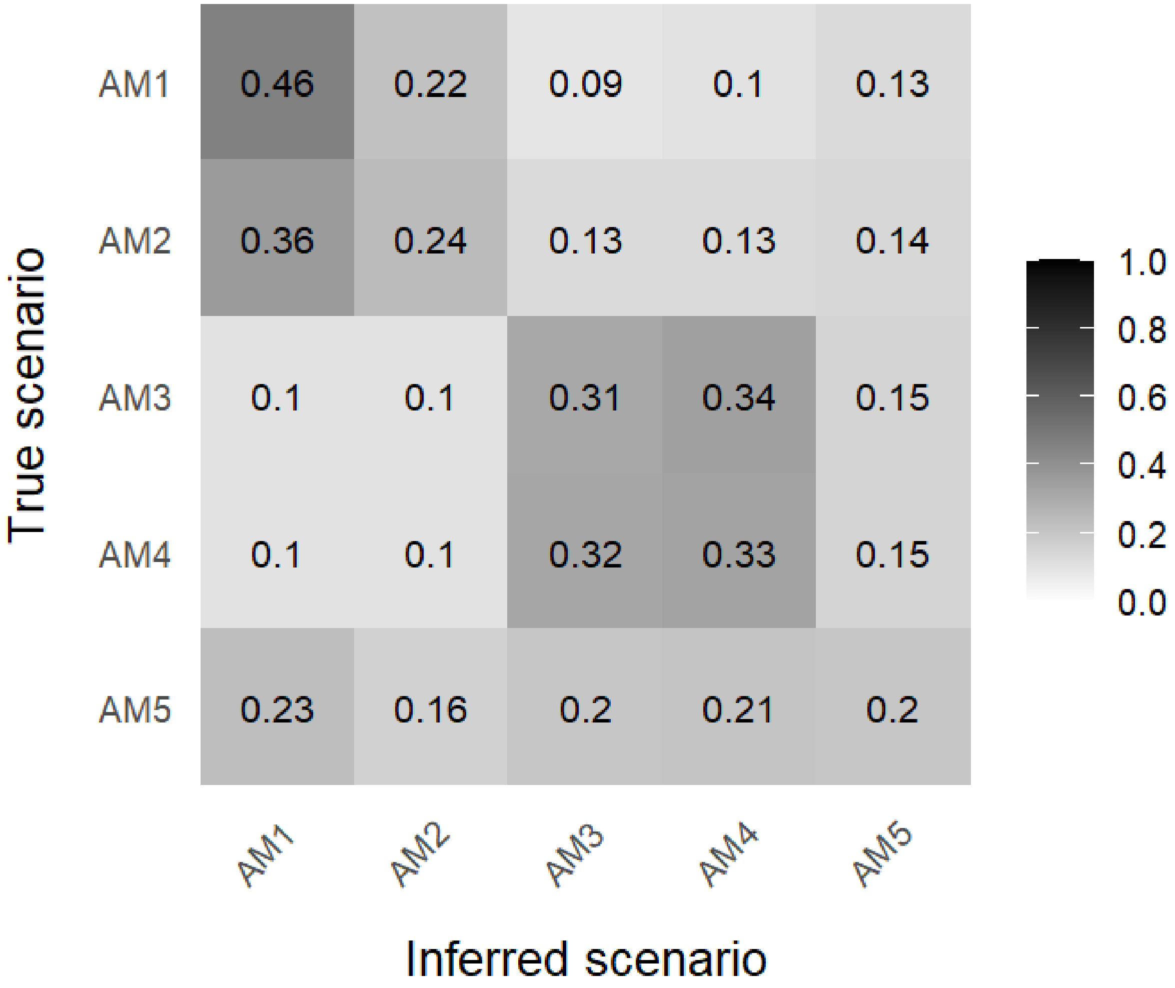


**Fig. S3. Confusion matrix of the theoretical investigation of five of the investigated scenarios (AM1-AM5) using PLS transformed pairwise values of inter- and intra-sample pseudo-haploid nucleotide diversity.** Calculation performed with abcrf R package (ABC-random forest approach, *80*), using 13,000 simulations per scenario and 2,000 trees. Each row represents simulations from a given scenario (the 'true' scenario), while the columns indicate the proportion of those simulations that ABC attributed to each of the studied scenarios (the 'inferred' scenarios).

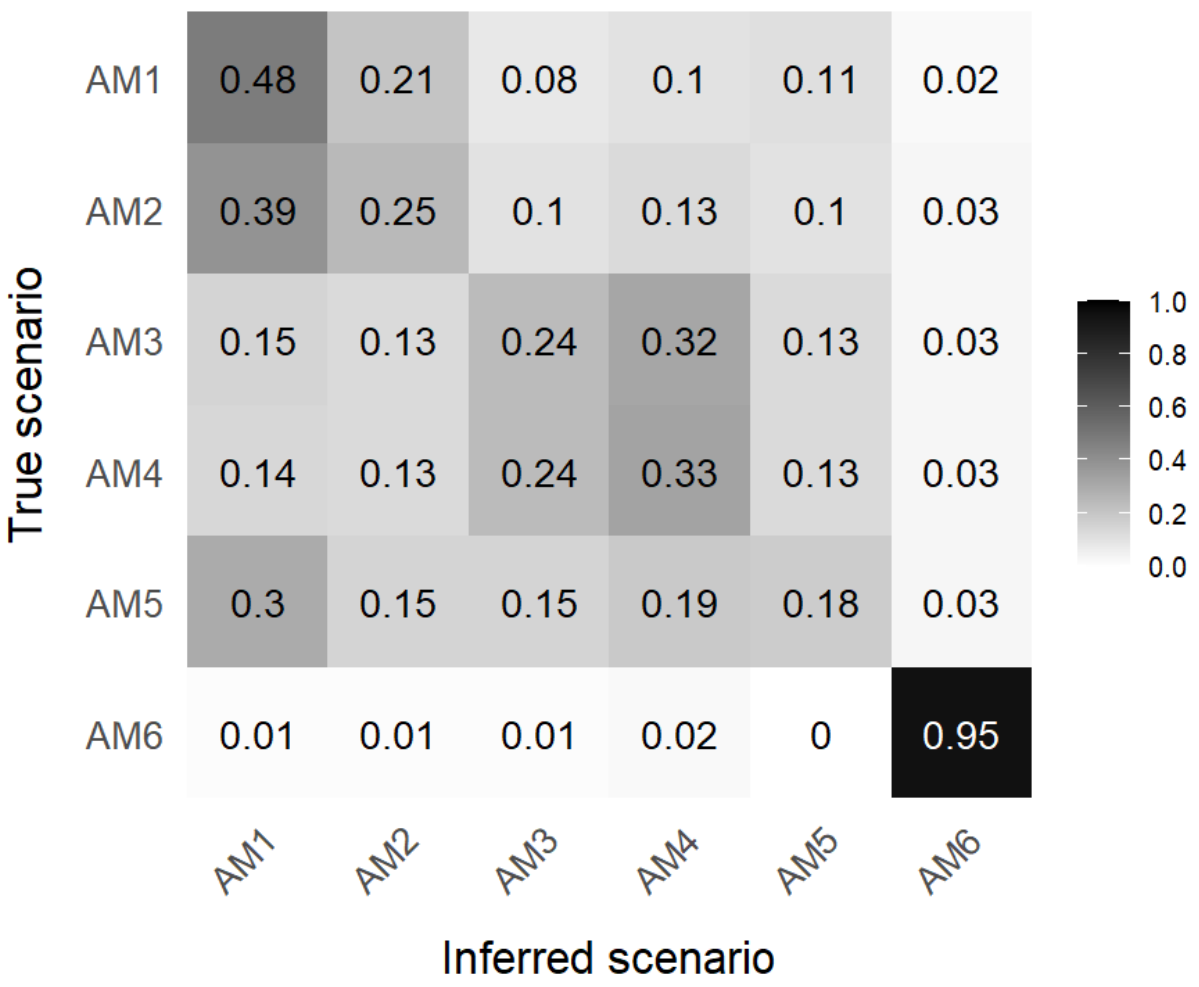


**Fig. S4. Graphical representation of the confusion matrix for the six investigated scenarios (AM1-AM6), without the genomes from Lepenski Vir.** Calculation performed with abcrf R package (ABC-random forest approach, *80*), using 33,500 simulations per scenario and 2,000 trees. Each row represents simulations from a given scenario (the 'true' scenario), while the columns indicate the proportion of those simulations that ABC attributed to each of the studied scenarios (the 'inferred' scenarios). For the analysis, the untransformed pairwise values of inter- and intra-sample pseudo-haploid nucleotide diversity were used.

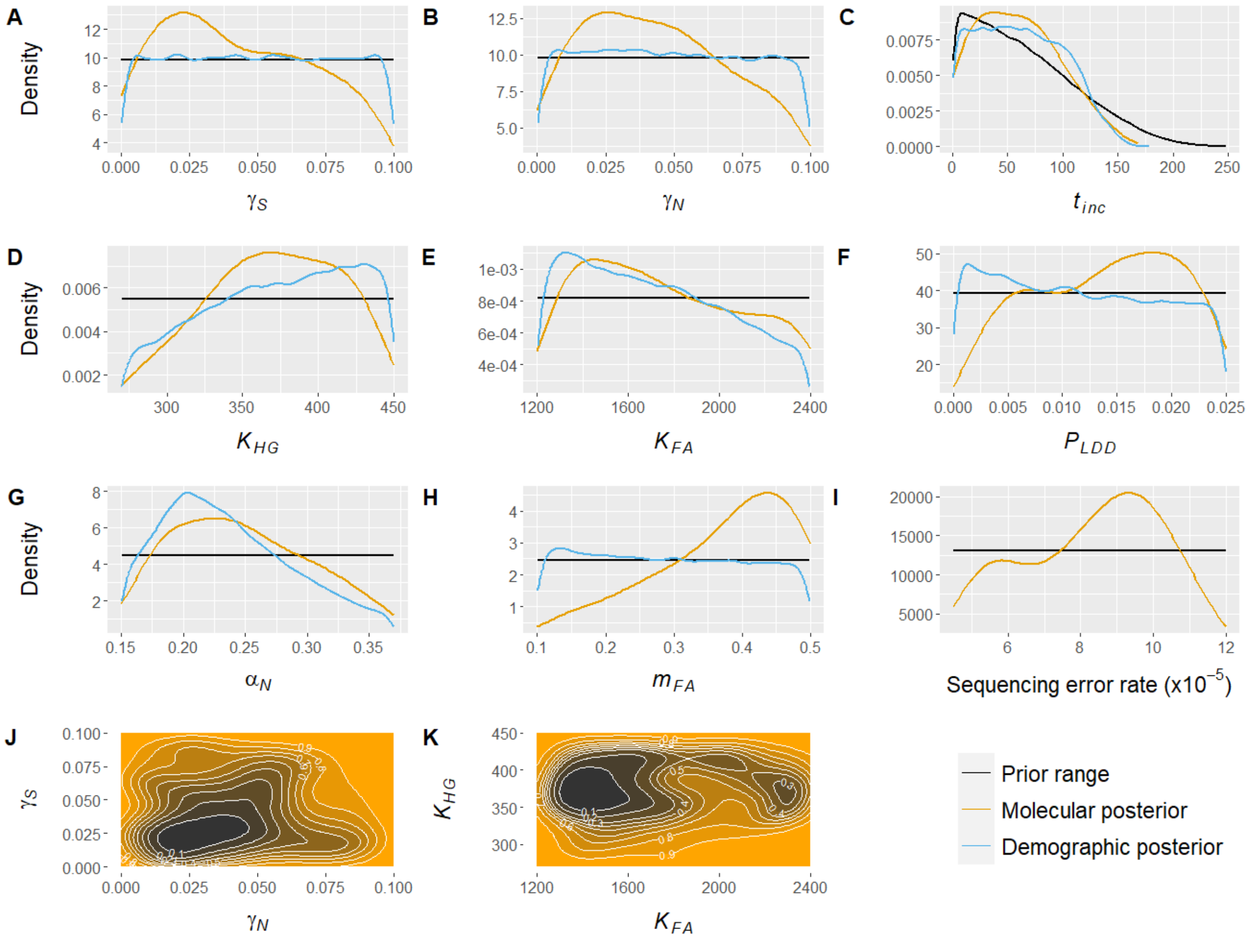


**Fig. S5. Posterior distribution of the estimated parameter values without the genomes from Lepenski Vir.** In panels A-I, the black line corresponds to the prior distribution, the light blue one to the distribution of parameter values of the simulations that were able to reproduce the observed dataset (demographic posterior), and the orange line corresponds to the posterior resulting from the ABC estimation (molecular posterior). **(A)** Admixture rate along the Southern Continental route ($\gamma_S$); **(B)** Admixture rate along the Northern Continental route ($\gamma_N$); **(C)** Number of generations during which the admixture rate increases ($t_{inc}$); **(D)** Effective population size of HGs ($K_{HG}$); **(E)** Effective population size of FAs ($K_{FA}$); **(F)** Proportion of FA migrations that are Long Distance Dispersals ($P_{LDD}$); **(G)** Competition coefficient along the Northern Continental route ($\alpha_N$); **(H)** Migration rate of FAs ($m_{FA}$); **(I)** Sequencing error rate (ε); **(J)** Two-dimensional posterior distribution of admixture rate along the Southern Continental route ($\gamma_S$) against the admixture rate along the Northern Continental route ($\gamma_N$); **(K)** Two-dimensional posterior distribution of effective population size of HGs ($K_{HG}$) against the Effective population size of FAs ($K_{FA}$). For the two-dimensional posterior distributions, orange represents the value combinations with lower probability and black the combinations with higher probability.

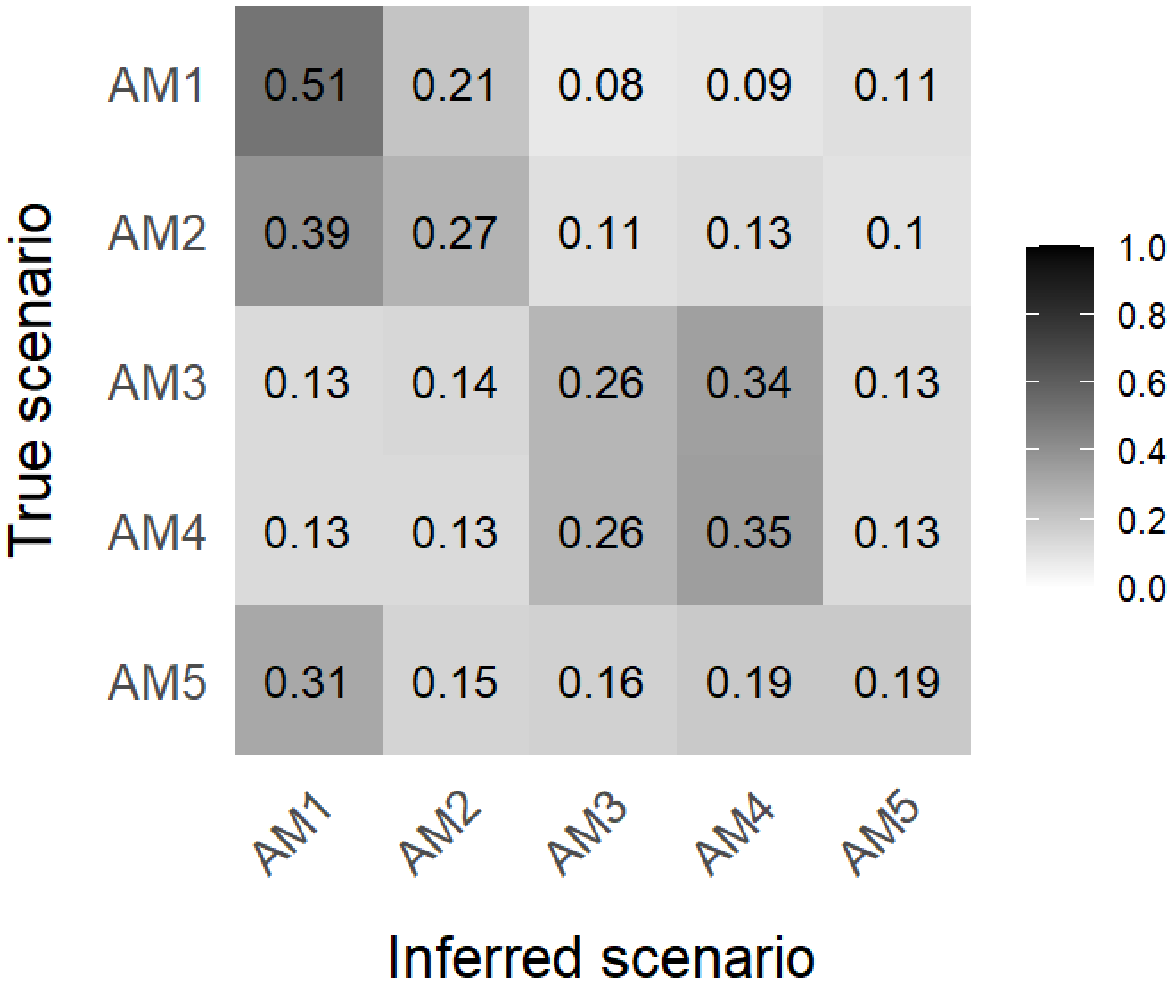


**Fig. S6. Confusion matrix for five of the investigated scenarios (AM1-AM5).** Calculation performed with abcrf R package (ABC-random forest approach, *80*), using 33,500 simulations per scenario and 2,000 trees. Each row represents simulations from a given scenario (the 'true' scenario), while the columns indicate the proportion of those simulations that ABC attributed to each of the studied scenarios (the 'inferred' scenarios). For the analysis, the untransformed pairwise values of inter- and intra-sample pseudo-haploid nucleotide diversity were used.

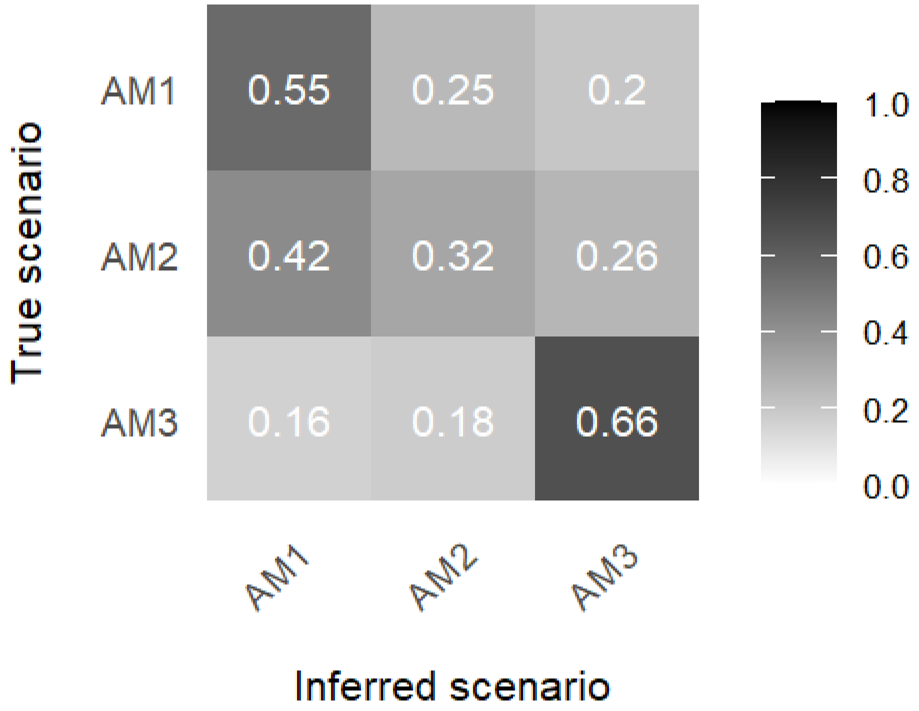


**Fig. S7. Graphical representation of the confusion matrix for three of the investigated scenarios (AM1-AM3).** Calculation performed with abcrf R package (ABC-random forest approach, *80*), using 33,500 simulations per scenario and 2,000 trees. Each row represents simulations from a given scenario (the 'true' scenario), while the columns indicate the proportion of those simulations that ABC attributed to each of the studied scenarios (the 'inferred' scenarios). For the analysis, the untransformed pairwise values of inter- and intra-sample pseudo-haploid nucleotide diversity were used.

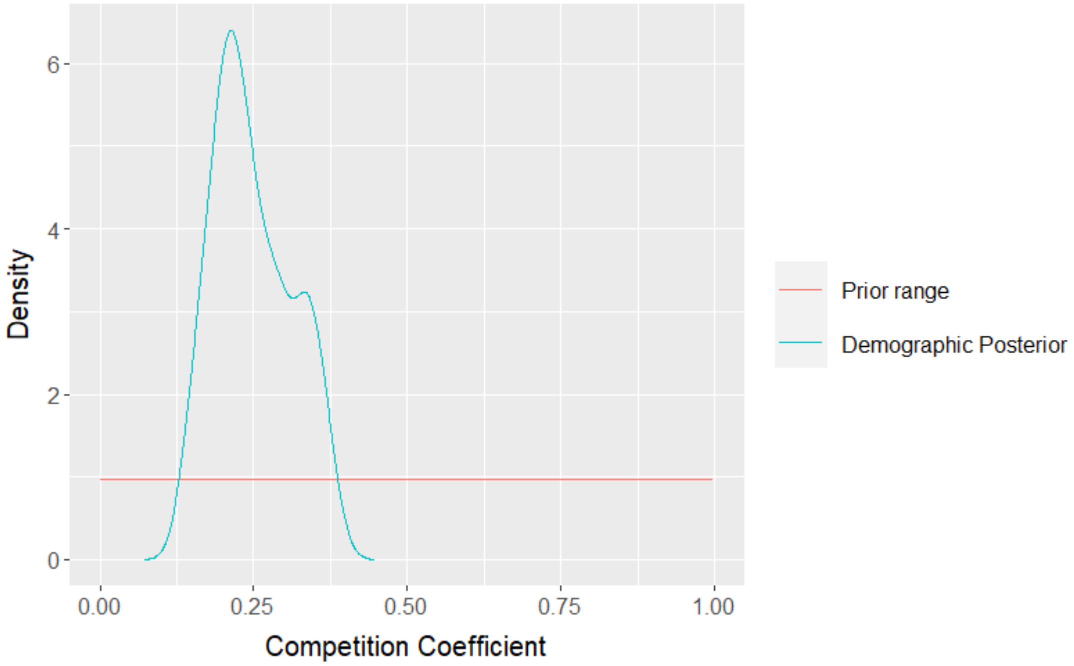


**Fig. S8. Posterior distribution of the values of the competition coefficient.** The red line corresponds to the prior distribution of values used for the simulations, while the light blue corresponds to the distribution of the values of the simulations that were able to reproduce the observed dataset.

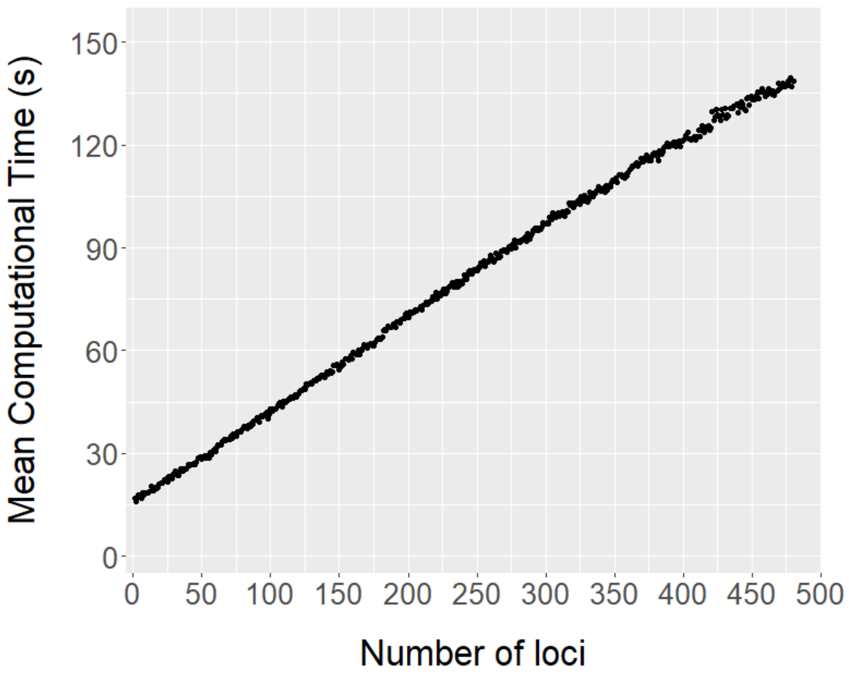


**Fig. S9. Computational time as a function of the number of simulated genetic loci.** Time is given in seconds and corresponds to the time required for the completion of one simulation. All simulations simulating the same number of genetic loci have been averaged to get the mean simulation time for each given number of loci.

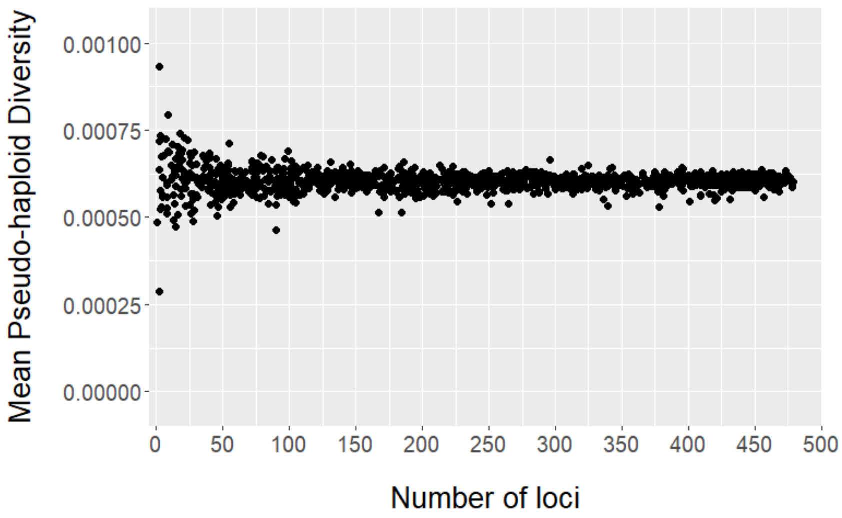


**Fig. S10. Effect of the number of simulated genetic loci on pseudo-haploid nucleotide diversity.** The simulated pseudo-haploid nucleotide diversity values of all simulations simulating the same number of genetic loci have been averaged to get the mean pseudo-haploid nucleotide diversity for each given number of loci.

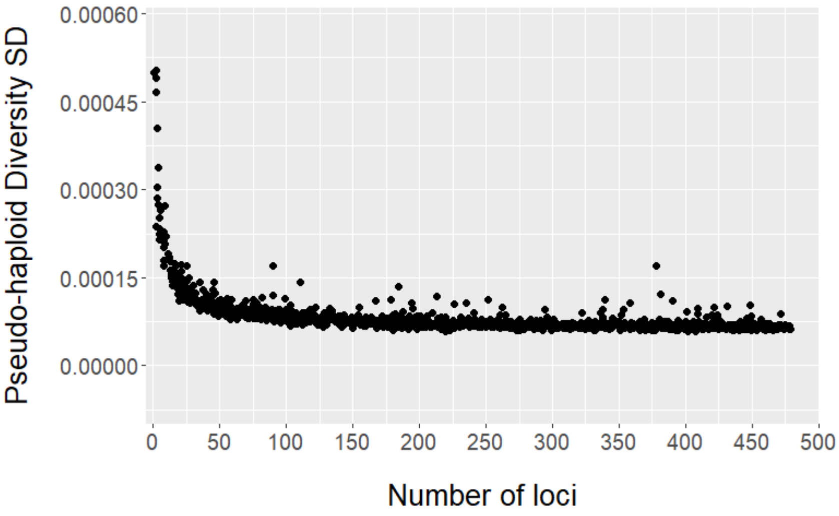


**Fig. S11. Effect of the number of simulated genetic loci on the variation of pseudo-haploid nucleotide diversity.** For each simulation, the standard deviation (SD) of the simulated pseudo-haploid nucleotide diversity was estimated. Then, the SD of all simulations simulating the same number of genetic loci was averaged to get the mean SD for each given number of loci.

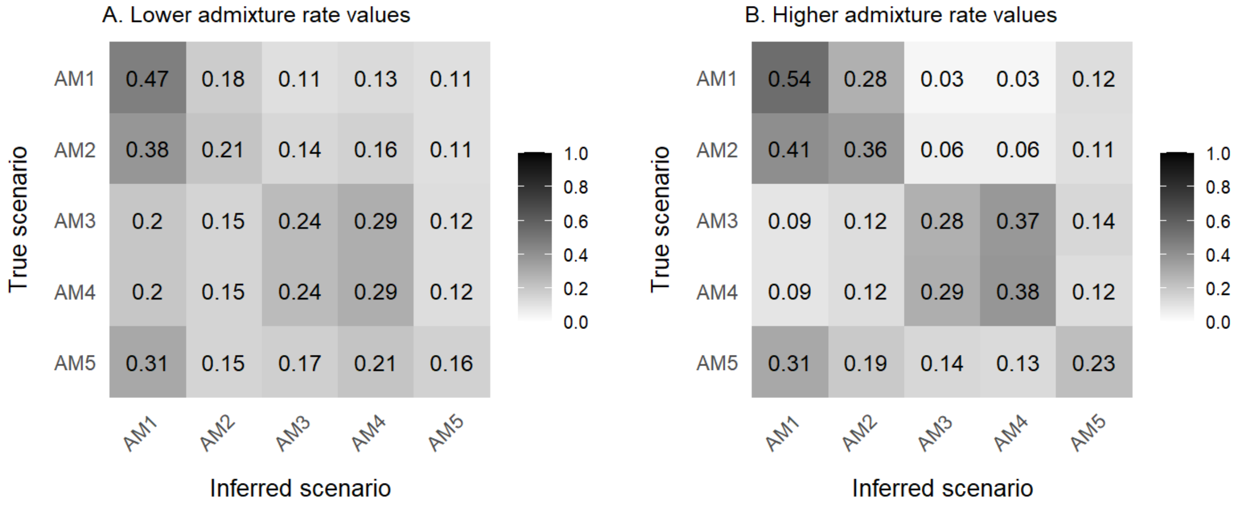


**Fig. S12. Confusion matrices of the ABC model choice for five of the investigated scenarios (AM1-AM5), performed on two different simulation sets.** A) Confusion matrix of the ABC model choice, using simulations with admixture rate within the lower half of the admixture rate prior range (0-0.05). B) Confusion matrix of the ABC model choice, using simulations with admixture rate within the upper half of the admixture rate prior range (0.05-0.1). Calculation performed with abcrf R package (random forest approach, *80*), using 16,750 simulations per scenario in each group and 2,000 trees. Each row represents simulations from a given scenario (the 'true' scenario), while the columns indicate the proportion of those simulations that ABC attributed to each of the studied scenarios (the 'inferred' scenarios). For the analysis, the untransformed pairwise values of inter- and intra-sample pseudo-haploid nucleotide diversity were used.

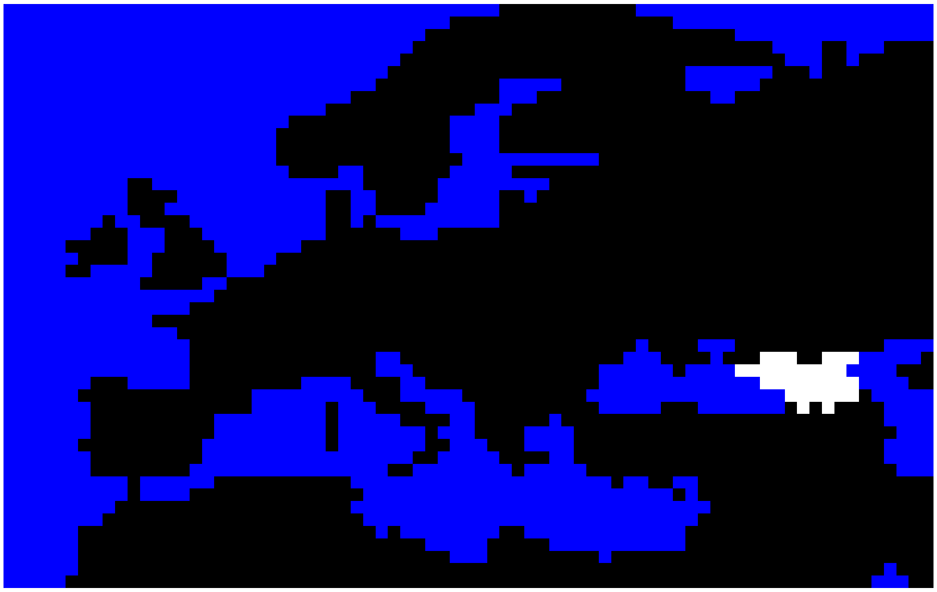

**Fig. S13. Graphical representation of the map used for simulating a scenario with a barrier in Caucasus.** The demes in black represent areas that can be inhabited and therefore migration can take place through them. The white demes represent an area that cannot be inhabited and therefore blocking migration.

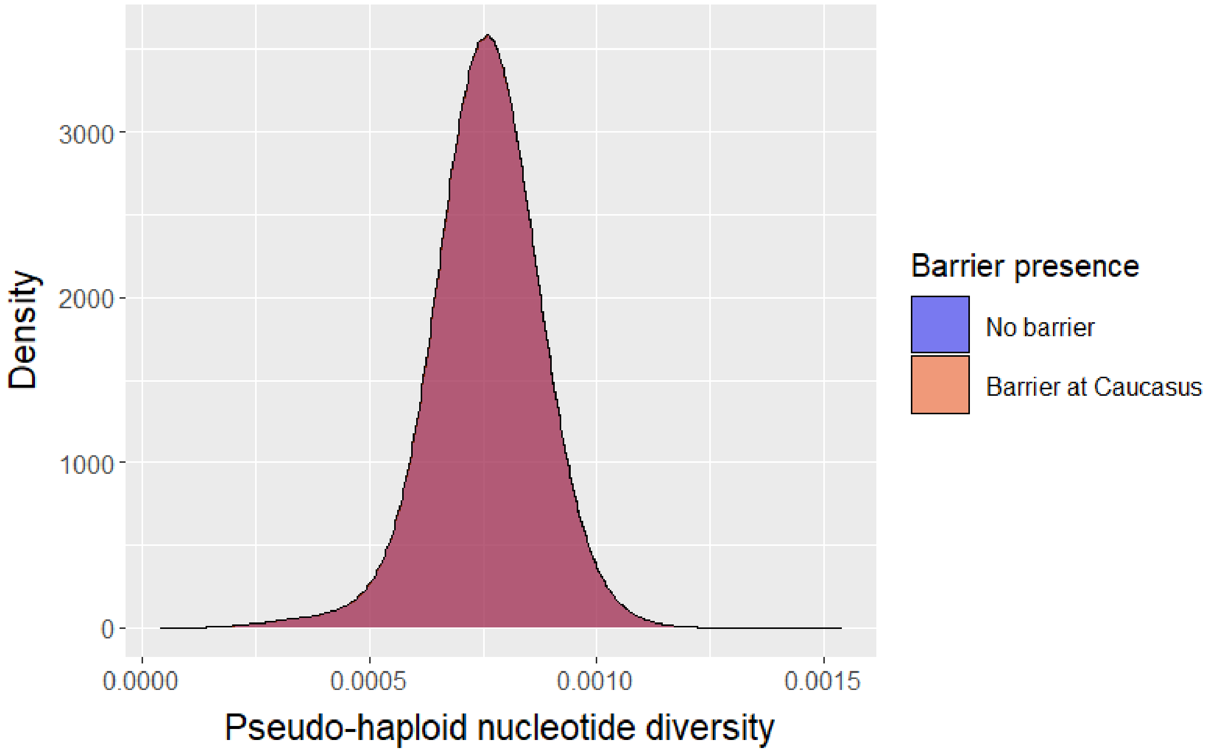


**Fig. S14. Distribution of the simulated pseudo-haploid nucleotide diversity values, for the investigation of the effect of a geographical barrier.** In the first scenario the movement of individuals through Caucasus is unimpeded, while in the simulations of the second scenario a geographical barrier is present in Caucasus, preventing the migration through this area.

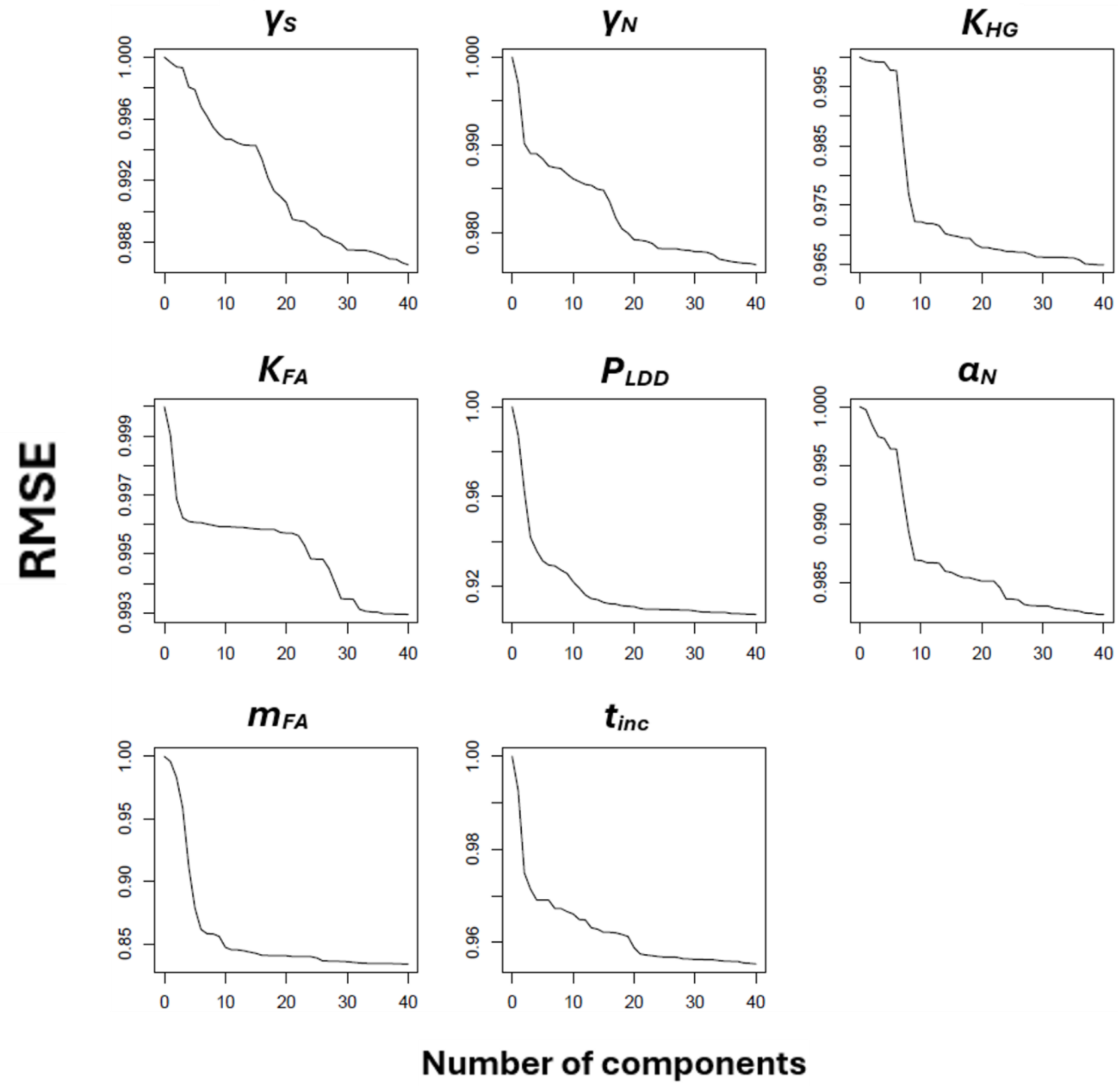


**Fig. S15. Root mean square error (RMSE) as a function of the number of PLS components for each estimated parameter.** The PLS regression and the estimation of the RMSE were performed with the "find_pls.R" R script included in ABCtoolbox2 (*81*). $\boldsymbol{\gamma_S}$: admixture rate along the Southern Continental route; $\boldsymbol{\gamma_N}$: admixture rate along the Northern Continental route; $\boldsymbol{K_{HG}}$: effective population size of Hunter-Gatherers; $\boldsymbol{K_{FA}}$: effective population size of Farmers; $\boldsymbol{P_{LDD}}$: proportion of FA migrations that are Long Distance Dispersals; $\boldsymbol{\alpha_N}$: competition coefficient along the Northern Continental route; $\boldsymbol{m_{FA}}$: migration rate of FAs; $\boldsymbol{t_{inc}}$: number of generations during which the admixture rate increases.

**Table S1. Marginal posterior density p-values of the six investigated scenarios.** Calculation performed with ABCtoolbox2 (*81*), using 33,500 simulations per scenario and three separate tolerance levels, retaining 500, 1000 and 1,675 simulations (tolerance of 0.015, 0.03 and 0.05 respectively). For this analysis, the observed values of pseudo-haploid nucleotide diversity were transformed into PLS components.

| | Marginal posterior density p-value with tolerance 0.015 | Marginal posterior density p-value with tolerance 0.03 | Marginal posterior density p-value with tolerance 0.05 |
|---|---|---|---|
| AM1 - Constant admixture rate in space and time | 0.45 | 0.31 | 0.91 |
| AM2 - Spatially increasing admixture rate | 0.12 | 0.65 | 1.00 |
| AM3 - Temporally increasing admixture rate | 0.46 | 0.89 | 0.28 |
| AM4 - Spatially and temporally increasing admixture rate | 0.39 | 0.67 | 0.46 |
| AM5 - Spatially decreasing admixture rate | 1.00 | 0.24 | 0.82 |
| AM6 - Variable duration of admixture rate increase | 0.39 | 0.35 | 0.23 |

**Table S2. Model choice between the six investigated scenarios.** Performed with abcrf R package (ABC-random forest approach, *80*), using 2,000 trees and 33,500 simulations per scenario. The votes refer to the number of trees that selected each scenario as the most probable one. For the analysis, the untransformed summary statistics were used.

| AM1 Constant admixture rate in space and time votes | AM2 Spatially increasing admixture rate votes | AM3 Temporally increasing admixture rate votes | AM4 Spatially and temporally increasing admixture rate votes | AM5 Spatially decreasing admixture rate | AM6 Variable duration of admixture rate increase | Chosen scenario | Posterior probability of chosen scenario |
|---|---|---|---|---|---|---|---|
| 156 | 129 | 171 | 177 | 162 | 1205 | AM6 | 0.77 |

**Table S3. Marginal posterior density p-values of the six investigated scenarios without genomes from Lepenski Vir.** Calculation performed with ABCtoolbox2 (*81*), using 33,500 simulations per scenario and a tolerance level of 0.03, retaining 1,000 simulations. For this analysis the observed values of pseudo-haploid nucleotide diversity were transformed into PLS components.

| | Marginal posterior density p-value with tolerance 0.03 |
|---|---|
| AM1 - Constant admixture rate in space and time | 0.92 |
| AM2 - Spatially increasing admixture rate | 0.26 |
| AM3 - Temporally increasing admixture rate | 1 |
| AM4 - Spatially and temporally increasing admixture rate | 0.83 |
| AM5 - Spatially decreasing admixture rate | 0.56 |
| AM6 - Variable duration of admixture rate increase | 0.08 |

**Table S4. Model choice between the six investigated scenarios without the genomes from Lepenski Vir.** Performed with abcrf R package (ABC-random forest approach, *80*), using 2,000 trees and 33,500 simulations per scenario. The votes refer to the number of trees that selected each scenario as the most probable one. For the analysis, the untransformed pairwise values of inter- and intra-sample pseudo-haploid nucleotide diversity were used.

| AM1 Constant admixture rate in space and time votes | AM2 Spatially increasing admixture rate votes | AM3 Temporally increasing admixture rate votes | AM4 Spatially and temporally increasing admixture rate votes | AM5 Spatially decreasing admixture rate | AM6 Variable duration of admixture rate increase | Chosen scenario | Posterior probability of chosen scenario |
|---|---|---|---|---|---|---|---|
| 118 | 119 | 164 | 205 | 136 | 1258 | AM6 | 0.70 |

**Table S5. Characteristics of the estimated parameters, using the simulations of scenario AM6 without the genomes from Lepenski Vir.** A tolerance level of 0.01 was used, retaining 1,000 simulations out of the 100,000 simulations of the scenario. The estimation was performed with ABCtoolbox2 (*81*).

| Parameter | Posterior Mode | Posterior Mode bias | Posterior Mean | Posterior Mean bias | Posterior Lower 95% HDI | Posterior Upper 95% HDI |
|---|---|---|---|---|---|---|
| Admixture rate in Southern Continental route ($\gamma_S$) | 0.022 | 3.44 | 0.045 | 4.41 | 0 | 0.091 |
| Admixture rate in Northern Continental route ($\gamma_N$) | 0.025 | 2.2 | 0.045 | 2.8 | 0.0005 | 0.091 |
| Number of generations of $\gamma$ increase ($t_{inc}$) | 36 | 1.35 | 62 | 1.82 | 1 | 129 |
| Effective population size of HGs ($K_{HG}$) | 368 | 0.14 | 369 | 0.11 | 290 | 447 |
| Effective population size of FAs ($K_{FA}$) | 1442 | 0.18 | 1760 | 0.17 | 1230 | 2341 |
| Migration rate of FAs ($m_{FA}$) | 0.436 | 0.41 | 0.36 | 0.42 | 0.17 | 0.5 |
| Proportion of Long-Distance Dispersals ($P_{LDD}$) | 0.018 | 1.52 | 0.013 | 1.94 | 0.002 | 0.025 |
| Coefficient of competition in Northern Continental route ($\alpha_N$) | 0.226 | 0.18 | 0.248 | 0.19 | 0.154 | 0.347 |
| Sequencing Error rate ($\varepsilon$) | 0.00001 | 0.22 | 0.00008 | 0.2 | 0.000047 | 0.00011 |

**Table S6. Model choice between five of the investigated scenarios (AM1-AM5).** Performed with abcrf R package (ABC-random forest approach, *80*), using 2,000 trees and 33,500 simulations per scenario. The votes refer to the number of trees that selected each scenario as the most probable one. For the analysis, the untransformed summary statistics were used.

| AM1 Constant admixture rate in space and time votes | AM2 Spatially increasing admixture rate votes | AM3 Temporally increasing admixture rate votes | AM4 Spatially and temporally increasing admixture rate votes | AM5 Spatially decreasing admixture rate | Chosen scenario | Posterior probability of chosen scenario |
|---|---|---|---|---|---|---|
| 363 | 403 | 446 | 430 | 358 | AM3 | 0.43 |

**Table S7. Model choice performed on three of the investigated scenarios (AM1-AM3).** Performed with abcrf R package (ABC-random forest approach, *80*), using 2,000 trees and 33,500 simulations per scenario. The votes refer to the number of trees that selected each scenario as the most probable one. For the analysis, the untransformed pairwise values of inter- and intra-sample pseudo-haploid nucleotide diversity were used.

| AM1 - Constant admixture rate in space and time votes | AM2 - Spatially increasing admixture rate votes | AM3 - Temporally increasing admixture rate votes | Chosen scenario | Posterior probability of chosen scenario |
|---|---|---|---|---|
| 630 | 660 | 710 | AM3 | 0.52 |

**Table S8. Characteristics of the estimated parameters for two additional tolerance levels, using the simulations of scenario AM6.** The tolerance levels of 0.005 and 0.05 correspond to 500 and 5,000 retained simulations out of the 100,000 simulations of the scenario. The estimation was performed with ABCtoolbox2 (*81*).

| Parameter | Tolerance level | Posterior Mode | Posterior Mode relative bias | Posterior Mean | Posterior Mean relative bias | Posterior Lower 95% HDI | Posterior Upper 95% HDI |
|---|---|---|---|---|---|---|---|
| Admixture rate in Southeast Continental route ($\gamma_S$) | 0.005 | 0.029 | 4.64 | 0.047 | 4.13 | 0.002 | 0.092 |
| | 0.05 | 0.029 | 3.33 | 0.049 | 3.19 | 0.004 | 0.096 |
| Admixture rate in Northwest Continental route ($\gamma_N$) | 0.005 | 0.035 | 3.25 | 0.049 | 2.73 | 0.004 | 0.096 |
| | 0.05 | 0.041 | 2.79 | 0.049 | 3.09 | 0.003 | 0.094 |
| Number of generations of $\gamma$ increase ($t_{inc}$) | 0.005 | 60 | 1.35 | 72 | 2.18 | 3 | 137 |
| | 0.05 | 55 | 1.4 | 65 | 2.2 | 1 | 129 |
| HG effective population size ($K_{HG}$) | 0.005 | 335 | 0.13 | 355 | 0.11 | 275 | 433 |
| | 0.05 | 337 | 0.14 | 354 | 0.12 | 272 | 435 |
| FA effective population size ($K_{FA}$) | 0.005 | 1430 | 0.19 | 1763 | 0.17 | 1230 | 2332 |
| | 0.05 | 1467 | 0.18 | 1736 | 0.17 | 1200 | 2269 |
| Migration rate of FAs ($m_{FA}$) | 0.005 | 0.021 | 2.87 | 0.014 | 4.27 | 0.002 | 0.025 |
| | 0.05 | 0.016 | 1.81 | 0.013 | 2.63 | 0.002 | 0.024 |
| Proportion of Long-Distance Dispersals ($P_{LDD}$) | 0.005 | 0.214 | 0.17 | 0.24 | 0.19 | 0.15 | 0.336 |
| | 0.05 | 0.199 | 0.18 | 0.227 | 0.19 | 0.15 | 0.321 |
| Competition coefficient in Northwest Continental route ($\alpha_N$) | 0.005 | 0.411 | 0.38 | 0.34 | 0.36 | 0.163 | 0.5 |
| | 0.05 | 0.427 | 0.41 | 0.339 | 0.4 | 0.153 | 0.5 |
| Sequencing Error rate ($\varepsilon$) | 0.005 | 0.000059 | 0.216 | 0.000074 | 0.222 | 0.000045 | 0.000107 |
| | 0.05 | 0.000056 | 0.207 | 0.000071 | 0.216 | 0.000045 | 0.000102 |

**Table S9. Equation for the implementation of sequencing errors.** This equation is used to compute the probability $P_{nodiff}$ to find no difference at the pseudo-haploid level between two genomes with known diploid state, given a sequencing error rate $\varepsilon$ (using the majority allele and assuming a depth >2).

| Diploid state | 00 | 01 | 11 |
|---|---|---|---|
| 00 | $P_{nodiff}= \varepsilon^2 + (1-\varepsilon)^2$ | $P_{nodiff}=1/2$ | $P_{nodiff}=2\varepsilon(1-\varepsilon)$ |
| 01 | $P_{nodiff}=1/2$ | $P_{nodiff}=1/2$ | $P_{nodiff}=1/2$ |
| 11 | $P_{nodiff}= 2\varepsilon(1-\varepsilon)$ | $P_{nodiff}=1/2$ | $P_{nodiff}=\varepsilon^2 + (1-\varepsilon)^2$ |

**Data S1. (separate file)**
**Information of the genomic data used in the present study.** The information provided are: the name assigned to the individual (Individual_ID); if the individual was classified as being a hunter-gatherer (HG) or a farmer (FA) (Population_Group); the latitude of the area where the individual was found (Latitude); the longitude of the area where the individual was found (Longitude); the age of the individual in years before present (Mean_Age_YBP); the population sample to which the individual was assigned for estimating the mean intra- and inter-sample pseudo-haploid nucleotide diversity and performing the simulations (Sample); the study which produced the genome (Publication). The full references for the publications from which the genomes were retrieved are also provided.

**Data S2. (separate file)**
**Pairwise values of pseudo-haploid nucleotide diversity.** The values of pseudo-haploid nucleotide diversity for all genome pairs are provided.

**Data S3. (separate file)**

**Mean values of pseudo-haploid nucleotide diversity.** The mean values of intra- and inter-sample pseudo-haploid nucleotide diversity are provided. For creating the samples, genomes sampled in the same deme and at the same generation during the simulations were grouped together. The genomes belonging to each sample can be found in Data S1.
